# The Frequency of, and Factors Associated with Prolonged Hospitalization: A Multicentre Study in Victoria, Australia

**DOI:** 10.3390/jcm9093055

**Published:** 2020-09-22

**Authors:** Richard Ofori-Asenso, Danny Liew, Johan Mårtensson, Daryl Jones

**Affiliations:** 1 Department of Epidemiology and Preventive Medicine, School of Public Health and Preventive Medicine, Monash University, Melbourne, VIC 3800, Australia; asensox215@gmail.com (R.O.-A.); Danny.liew@monash.edu (D.L.); 2 Department of Pharmacy, Faculty of Health and Medical Sciences, University of Copenhagen, 1165 Copenhagen, Denmark; 3 Department of Physiology and Pharmacology, Section of Anaesthesia, and Intensive Care, Karolinska Institutet, 171 77 Stockholm, Sweden; johan.martensson@sll.se; 4 Department of Intensive Care Austin Hospital, Melbourne, VIC 3084, Australia

**Keywords:** hospitalization, bed utilization, mortality, length of stay

## Abstract

Background: Limited available evidence suggests that a small proportion of inpatients undergo prolonged hospitalization and use a disproportionate number of bed days. Understanding the factors contributing to prolonged hospitalization may improve patient care and reduce the length of stay in such patients. Methods: We undertook a retrospective cohort study of adult (≥20 years) patients admitted for at least 24 h between 14 November 2016 and 14 November 2018 to hospitals in Victoria, Australia. Data including baseline demographics, admitting specialty, survival status and discharge disposition were obtained from the Victorian Admission Episode Dataset. Multivariable logistic regression analysis was used to identify factors independently associated with prolonged hospitalization (≥14 days). Cox proportional hazard regression model was used to examine the association between various factors and in-hospital mortality. Results: There were almost 5 million hospital admissions over two years. After exclusions, 1,696,112 admissions lasting at least 24 h were included. Admissions with prolonged hospitalization comprised only 9.7% of admissions but utilized 44.2% of all hospital bed days. Factors independently associated with prolonged hospitalization included age, female gender, not being in a relationship, being a current smoker, level of co-morbidity, admission from another hospital, admission on the weekend, and the number of admissions in the prior 12 months. The in-hospital mortality rate was 5.0% for those with prolonged hospitalization compared with 1.8% in those without (*p* < 0.001). Prolonged hospitalization was also independently associated with a decreased likelihood of being discharged to home (OR 0.53, 95% CI 0.52–0.54). Conclusions: Patients experiencing prolonged hospitalization utilize a disproportionate proportion of bed days and are at higher risk of in-hospital death and discharge to destinations other than home. Further studies are required to identify modifiable factors contributing to prolonged hospitalization as well as the quality of end-of-life care in such admissions.

## 1. Introduction

There are competing demands for the provision of healthcare in modern hospitals of resource-rich countries. On the one hand, the population is ageing, and hospitalized patients have increased co-morbidity and complexity of disease [1,2,3,4]. Thus, hospitals continue to account for a significant proportion of health expenditure, with about two-fifths of health spending among The Organisation for Economic Co-operation and Development (OECD) countries devoted to hospital care [5].

On the other hand, there is a need to ensure the sustainability of hospitals by reducing hospital length of stay (LOS), optimizing utilisation of available resources, and minimizing the risk of nosocomial complications [6]. Studies have suggested that a significant proportion of inpatient days in modern hospitals are inappropriate [7,8]. Reducing medically inappropriate days in hospital could substantially lower the cost of providing hospital services [9,10]. Consequently, LOS has emerged as one of the key performance indicators in developed countries of hospital efficiency. In particular, improvements in LOS could free up beds and staff time and contribute to better provision and efficient use of hospital resources to address population health needs. 

Several studies have identified factors associated with protracted hospitalization, but many of these have focused on targeted patient populations [11,12,13,14,15,16,17,18,19,20,21,22,23,24,25,26,27,28,29,30,31,32,33,34,35,36,37,38,39,40] or involved data collected at a single centre [13,18,19,23,24,28,29,31,37,39,40,41,42,43,44,45] Understanding the factors that contribute to prolonged hospitalization in a broader patient cohort may guide the development of interventions that target at risk patients, improve overall care, and reduce resource utilisation. 

A recently published single-centre study from a large tertiary referral teaching hospital in Australia revealed that only 13.1% of hospital admissions lasted at least 14 days, but that such admissions accounted for 49.1% of all bed days [44]. This study identified several baseline factors associated with prolonged hospitalization and reported prolonged hospitalization to be associated with a 2.6-fold increased risk of in-hospital death [44]. Accordingly, we conducted a retrospective cohort study of all hospital admissions in the state of Victoria in Australia to evaluate the frequency of, and factors associated with prolonged hospitalization. In addition, we sought to determine if prolonged hospitalization was associated with an increased risk of in-hospital death. 

## 2. Methods

### 2.1. Ethics Approval

The conduct of the study was approved by the Monash University Human Research Ethics Committee (Project #: 14204), as well as the Victorian Department of Health and Human Services (DHHS) data privacy approver, as part of the data Custodian Approval process.

### 2.2. Study Design and Participants 

We conducted a retrospective cohort study using data collected between 14 November 2016 and 14 November 2018. We included all admission episodes (unit of analysis) involving patients aged ≥20 years admitted to acute care facilities across all of Victoria, Australia. We excluded same-day admissions (those lasting less than one calendar day), as well as admissions related to childbirth. In addition, we excluded patients with missing admission diagnoses, short term admissions related to dialysis, and patients who had an admission involving discharge to the “hospital in the home” service [46]. Patients who had an admission involving discharge to the “hospital in the home” service were excluded as they receive low level intensity of treatment in their own homes, often for extended periods. As the focus of the study was to examine utilization of acute hospital beds, such patients were not thought to be representative of acutely unwell hospitalized patients and may have skewed the overall proportion of patients with prolonged LOS.

### 2.3. Data Sources and Variables Collected 

Data were obtained from the Victorian Admissions Episodes Dataset (VAED) [46] and the Victorian Emergency Minimum Dataset (VEMD) [47]. These are administrative databases that capture all inpatient and emergency department (ED) encounters, respectively, for hospitals (public and private) in Victoria. Data are entered by dedicated and trained hospital staff and maintained by the Victorian DHHS. We extracted data related to the hospital type (private, regional, secondary, tertiary other as classified by the Victorian health authority), gender, age (provided in five-year age-groups), variables needed to calculate the Charlson co-morbidity index [48], smoking status, the number of hospital admissions in the past 12 months, Indigenous Australian status, marital status, emergency admission status, admission source, admission into the intensive care unit (ICU), and the time, day and season of admission. The length of stay used in the analysis was the duration of each hospital admission. If a patient was transferred from one hospital to another, then they had two records, each with an LOS. The second record would be coded as a transfer from another hospital. As the dataset did not allow for linkage of episodes of care provided at different hospitals in patients who were transferred, we adjusted for the source of admission (e.g., home, from another hospital, etc.). Private hospitals are defined as hospitals not funded or managed by the Victorian Department of health and human services. Publicly funded and managed hospitals include regional (country or non-metropolitan), secondary (medium sized metropolitan hospitals with limited specialty services) and tertiary (large University-affiliated hospitals with a wide range of specialty services). Admission diagnosis was also collected, based on the Australian refined diagnostic related group (DRG) classification of the primary diagnosis (AR-DRG v9.0) [46], and was categorized into one of 37 specialties based on the classification system used in the VAED (Supplementary Table S1) [46]. The 37 specialties were ranked and ordered according to the corresponding length of stay. They were then divided into four groups based on this ranking (numbered 1–4).

### 2.4. Outcomes

The primary outcomes (dependent variables) were prolonged hospitalization and in-hospital mortality. There is no accepted or consensus definition of what constitutes prolonged LOS. In keeping with other published studies [13,44], we chose a duration of 14 days. This period is relevant from both a clinical perspective and with regard to resource utilization. Importantly, it was defined *a priori*. Secondary outcomes were the proportion of overall bed days incurred by prolonged hospitalizations, as well as the proportion of patients discharged to home. The independent variables included in the logistic regression and Cox proportional models were the patients’ demographic and clinical parameters.

### 2.5. Statistical Analysis 

Descriptive statistics were used to summarise the baseline characteristics of the study population. A chi-squared test was used to compare all baseline categorical variables between patients who experienced prolonged hospitalization and those who did not, and those who died in hospital and those who survived. 

Univariate and multivariable logistic regression analyses were used to examine the associations between the various factors and prolonged length of hospitalization and discharge to home. In each of the multivariable analyses, all variables were entered simultaneously into the model. Additional sensitivity analysis was performed to confirm the various factors associated with prolonged hospitalization by restricting the analysis to only survivors.

In all models, multicollinearity was assessed using the variation inflation factor (VIF) in which values >3 indicates collinearity between the variables. No parameter was dropped as per the VIF values. The association between various factors and in-hospital mortality was assessed via Cox proportional hazards model. In both the logistic and Cox proportional hazards models, for age comparisons, a mid-point age (60–64 years) was selected as the reference group to distinguish older adults from younger individuals [49]. All analyses were performed using STATA 16/SE (StataCorp, College Station, TX, USA). A two-tailed *p*-value of <0.05 was considered statistically significant.

## 3. Results

### 3.1. Details of Study Cohort

**Figure 1:**
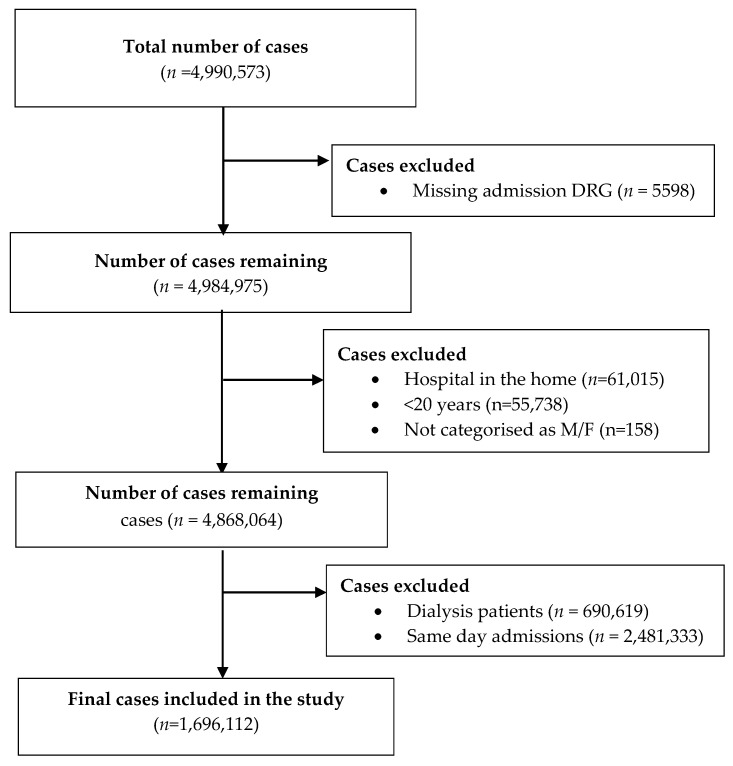
Details of multi-stay admissions included in the study following exclusion of admissions with missing data, same-day admissions, admissions associated with hospital in the home care, and patients less than 20 years. DRG = diagnostic related group, M = male, F = female.

During the two-year study period, there were 4.99 million admission cases. After exclusions, there were 1,696,112 admissions lasting at least 24 h (Figure 1). The median LOS was 3 (IQR 1–6) days. Overall, 51.7% of admissions occurred in females, and patients aged at least 65 years comprised 51.8% of the cohort. The majority (56.3%) of patients were in a relationship at the time of admission, and most were admitted from home (85.0%) and on weekdays (66.2%). Admissions were medical in 61.6% of cases and 49.5% were classified as emergency admissions (Table 1). 

### 3.2. Frequency of Prolonged Hospitalization and Details of Bed Utilisation 

The breakdown of hospital LOS and the corresponding utilisation of bed days is shown in Table 2. Admissions with prolonged hospitalization comprised only 9.7% of admissions, but utilized bed days, representing 44.2% of all hospital bed days for multi-day admissions (Table 2). 

### 3.3. Factors Associated with Prolonged Hospitalization

**Figure 2:**
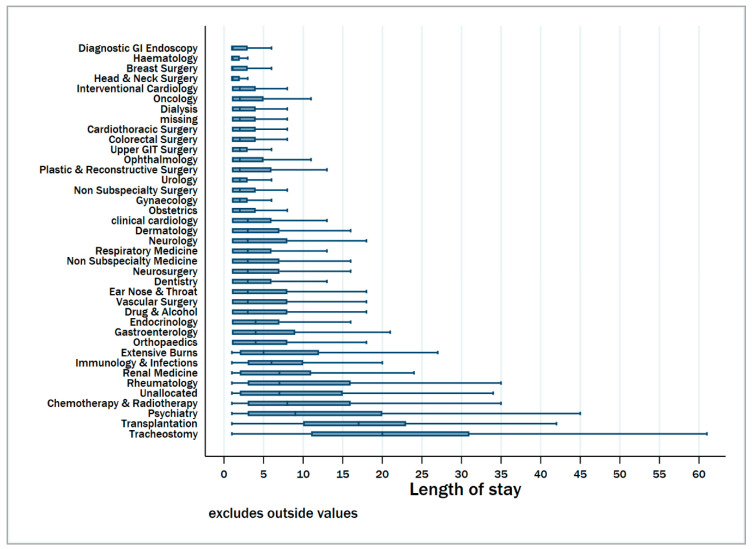
Boxplots showing median and inter-quartile length of hospital stay according to specialty services for patients hospitalized over two years in Victoria, Australia. Specialties are ranked according to increasing median length of stay. The risk categories were as follows. 1 (Diagnostic GI endoscopy-obstetrics); 2 (Clinical Cardiology-Drug & alcohol); 3 (Endocrinology-immunology & infections); 4 (Renal Medicine-Tracheostomy).

Univariable associations of different factors with prolonged hospitalization are presented in Supplementary Table S2. There were clear differences in the median hospital length of stay according to the specialty classification (Figure 2). From the multivariable analysis, factors independently associated with prolonged hospitalization included age above 65 years, female gender, not being in a relationship, being a current smoker, emergency (unscheduled) admission status, admission from another hospital, admission on a weekend, increasing number of admissions in the prior 12 months and admission into the ICU (Table 3). Moreover, patients admitted for surgical reasons were less likely to experience prolonged hospital stay compared to medical admissions. When restricting the analysis to only survivors, similar factors were determined to be associated with prolonged hospitalization (Supplementary Table S3).

### 3.4. Factors Associated with in-Hospital Mortality 

The in-hospital mortality rate for the overall cohort was 2.1%. It was 1.8% in patients who stayed for less than 14 days, and 5.0% for those with prolonged hospitalization (*p* < 0.001) (Supplementary Table S4). Factors independently associated with higher in-hospital mortality included increasing age, higher Charlson co-morbidity index and admission into a high-risk unit. Moreover, female gender was associated with a lower likelihood of in-hospital death (hazard ratio (HR) 0.90, 95% CI 0.88–0.92) whereas ICU stay (HR 3.17, 95% CI 3.06–3.28), and emergency (unscheduled) admission (HR 1.61, 95% CI 1.56–1.65) were also associated with increased risk of in-hospital death (Table 3).

### 3.5. Factors Associated with Hospital Discharge to Home 

Among survivors, 83.6% of patients were discharged to their private residence (Supplementary Table S5). Patients who experienced prolonged hospitalization were more likely to be discharged to a destination other than home compared with those without a prolonged hospitalization (36.5% vs. 14.4%; *p* < 0.001). After adjusting for other co-variates, prolonged hospitalization was associated with a decreased likelihood of being discharged to home (OR 0.53, 95% CI (0.52–0.54)); (Supplementary Table S6). Moreover, other factors predicting admission to home included age, gender, admission source, ICU stay, season, and the risk category of the admitting unit.

## 4. Discussion

### 4.1. Key Findings 

We conducted a retrospective cohort study of adult inpatients admitted for at least 24 h in Victoria over a two-year period to better understand the epidemiology of prolonged hospital admissions. We found that prolonged hospitalization comprised only one-tenth of multi-day admissions but accounted for nearly half of all bed days. Long stay patients were older, had greater co-morbidity, were more frequently admitted in the prior 12 months, and were more likely to be admitted under a medical unit. Moreover, such patients were more likely to die in hospital, and less likely to go home. 

### 4.2. Comparison with Previous Studies 

Most previous studies of prolonged hospitalization have focused on specific sub-groups of patients such as orthopaedic procedures [21,24,26,29,30,31,39], cardiovascular disease [13,15,17,36,37,38], solid organ transplantation [33,50], or oncological conditions [11,14,18,20,27,28,34,35] (Supplementary Table S7). A limited number of studies have examined the epidemiology of prolonged hospitalization in internal medicine patients [23,45,51] but only one of these was multi-centre in nature [51]. Our study is one of few large multi-centre studies examining the characteristics of, and factors associated with prolonged hospitalization in a heterogenous population of patients.

In accordance with two previous studies, we defined prolonged hospitalization as an admission lasting at least 14 days [13,44]. This period is relevant from both a clinical perspective and in terms of healthcare utilization. Importantly, this cutoff was chosen a priori. Other studies have variably defined prolonged hospitalization as admissions lasting more than seven days [19,23,29,37,38] or above the 75th centile for length of stay [11,12,14,15,17,20,26,27,30,40].

Relatively few studies have examined the proportion of overall bed days used by patients experiencing prolonged hospitalization. Using the same definition for prolonged hospitalization, O’Sullivan et al. conducted a single-centre study within the same jurisdiction as our study and similarly found that 13.1% of hospital admissions were at least 14 days, but used 49.1% of bed days [44]. Marfil-Garza et al. conducted a single-centre study in Mexico and found that 5.1% of admissions had a LOS more than 34 days but used 23.1% of total bed days [42]. Among general medicine discharges, Anderson et al. revealed that 2.3% of discharges were longer than 21 days, but accounted for 18.6% of total inpatient days [45]. 

Our study identified several independent baseline associations with prolonged hospitalization. Other studies have similarly revealed that increasing age [12,17,18,20,22,24,25,28,29,30,33,34,36,38,39,40,50], female gender [22,24,26,30,36,39,50], not being in a relationship, [52] being a current smoker [39], level of co-morbidity [11,12,14,15,16,18,22,26,40,42,44], weekend admissions [42], and the number of prior admissions [25,32,44] were associated with prolonged hospitalization. 

Finally, we found that prolonged hospitalization was independently associated with an increased risk of in-hospital death as well as a decreased likelihood of being discharged to home. Similar findings were found in the study by O’Sullivan et al. [44], which reported that prolonged hospitalization was associated with a 2.6-fold increase in in-hospital death and a lower likelihood of discharge to home, and Marfil-Garza et al. [42], who revealed a 3.6 fold increase in mortality in long-stay patients.

### 4.3. Study Strengths and Limitations 

The strengths of our study include the large cohort that was complete for all of Victoria, adjustment for multiple co-variates and identification of readily measured baseline co-variates that are independently associated with prolonged hospitalization. 

Limitations include the use of coding information and lack of information on advance care planning or end-of-life care. Specifically, many inpatients being palliated would have incurred longer LOS, and hence the association between prolonged hospitalization and in-hospital death was likely to have been bi-directionally causal. In addition, we cannot comment about the preventability of either the prolonged hospitalization or the associated increased risk of in-hospital death. It is possible that such patients may have not responded to appropriate treatment or alternatively, may have errors in diagnosis and/or treatment. Our study did not address specific diagnoses that contributed to prolonged LOS, which would be useful to policy-makers to decrease healthcare-related costs. However, our study does identify the groups in which interventions might be focused. For example, we found that patients who experience recurrent admissions and those not in a relationship are at increased risk of prolonged hospitalization. This suggests that increasing social supports in the community may reduce readmissions and/or timeliness of hospital discharge. The state of Victoria has a well-established primary health care network, and all hospitals have on-site outpatient facilities for review of patients following discharge. Enhancing the use of these services, as well as the interface between hospital and primary care, is likely to lead to reductions in hospital LOS and re-admissions. 

Finally, the observed increase in mortality associated with prolonged hospitalization may, in part, relate to a longer period of subject surveillance. We are not able to comment on whether prolonged hospitalization was associated with an increased risk of mortality beyond hospital discharge. 

### 4.4. Implications for Clinicians and Policy-Makers 

We have found several associations between baseline patient characteristics and care delivery factors and prolonged hospitalization. While most of these factors are not modifiable, our findings permit the identification of patients at risk of prolonged hospitalization, who may be the target of interventions.

Patients experiencing prolonged hospitalization utilized four million bed days in Victoria over a two-year period. Even a reduction of the hospital length of stay by just 5% in such patients would save more than 104 thousand bed days annually in Victoria.

Our analysis has revealed that patients experiencing prolonged hospitalization experienced more frequent hospital admissions in the prior 12 months. Improvements in outpatient or primary care may assist in preventing some of these admissions or shorten their duration. In addition, such patients were more likely to require rehabilitation or to be discharged to residential care. Improved access to such facilities may correspondingly reduce the LOS in the acute care facility. 

We also revealed that prolonged hospitalization was associated with an increased risk of death. It is possible that improved advance care planning and end-of-life care in this co-morbid, mostly medical, cohort may reduce hospital resource utilisation. 

### 4.5. Areas for Future Research 

It is important to conduct further research to identify the clinical and process-related factors that are contributing to patients remaining in hospital, particularly those that may be modifiable. In addition, it will be important to evaluate whether there is sub-optimal end-of-life care and continuation of non-beneficial treatments in patients who experience prolonged hospitalization, particularly those who die in hospital. 

## 5. Conclusions

Patients experiencing prolonged hospitalization comprise approximately one-tenth of multi-day admissions but utilize four-tenths of bed days. Such patients have different baseline characteristics and are at increased risk of in-hospital mortality. Further research is needed to identify factors contributing to prolonged hospitalization to improve patient outcomes and reduce resource utilization.

## Figures and Tables

**Table 1:** Characteristics of patients hospitalized over two years in Victoria, Australia.

Variable	Overall	Period	
		14 Nov. 2016–13 Nov. 2017	14 Nov. 2017–14 Nov. 2018
**Number of cases (*n*)**	1,696,112	834,045	862,067
Female (*n*, %)	877,020 (51.7)	431,543 (51.7)	445,477 (51.7)
Age groups, years. (*n*, %)			
20–24	61,465 (3.6)	30,666 (3.7)	30,799 (3.6)
25–29	65,547 (3.9)	32,465 (3.9)	33,082 (3.8)
30–34	72,766 (4.3)	35,991 (4.3)	36,775 (4.3)
35–39	75,571 (4.5)	37,261 (4.5)	38,310 (4.4)
40–44	82,135 (4.8)	41,519 (5.0)	40,616 (4.7)
45–49	95,566 (5.6)	47,104 (5.7)	48,462 (5.6)
50–54	103,747 (6.1)	51,793 (6.2)	51,954 (6.0)
55–59	121,095 (7.1)	59,470 (7.1)	61,625 (7.2)
60–64	137,130 (8.1)	67,467 (8.1)	69,663 (8.1)
65–69	163,254 (9.6)	81,217 (9.7)	82,037 (9.5)
70–74	170,359 (10.0)	81,641 (9.8)	88,718 (10.3)
75–79	168,124 (9.9)	82,130 (9.9)	85,994 (10.0)
80–84	158,335 (9.3)	77,411 (9.3)	80,924 (9.4)
≥85	221,018 (13.0)	107,910 (12.9)	113,108 (13.1)
Indigenous population (*n*, %)	13,888 (0.8)	6604 (0.8)	7284 (0.8)
Marital Status (*n*, %)			
Never married	325,672 (19.2)	160,081 (19.2)	165,591 (19.2)
Widowed	244,190 (14.4)	120,811 (14.5)	123,379 (14.3)
Married or defacto	955,280 (56.3)	470,118 (56.4)	485,162 (56.3)
Divorced or separated	143,222 (8.4)	69,762 (8.4)	73,460 (8.5)
Other	27,748 (1.6)	13,273 (1.6)	14,475 (1.7)
DRG type			
Medical	1,044,369 (61.6)	508,577 (61.0)	535,792 (62.2)
Surgical	571,449 (33.7)	285,911 (34.3)	285,538 (33.1)
Other	80,294 (4.7)	39,557 (4.7)	40,737 (4.7)
Emergency admission (*n*, %)	839,360 (49.5)	405,571 (48.6)	433,789 (50.3)
Admission source (*n*, %)			
Home	1,442,065 (85.0)	710,111 (85.1)	731,954 (84.9)
Aged care residential facility	11,302 (0.7)	5234 (0.6)	6068 (0.7)
From another hospital	195,230 (11.5)	95,892 (11.5)	99,338 (11.5)
Other	47,515 (2.8)	22,808 (2.7)	24,707 (2.9)
ICU stay (*n*, %)	79,115 (4.7)	39,187 (4.7)	39,928 (4.6)
Admitted on weekend (*n*, %)	573,292 (33.8)	282,965 (33.9)	290,327 (33.7)
Season of admission (*n*, %)			
Spring (Sep-Nov)	406,209 (24.0)	204,835 (24.6)	201,374 (23.4)
Summer (Dec-Feb)	415,172 (24.5)	203,530 (24.4)	211,642 (24.6)
Autumn (Mar-May)	433,328 (25.6)	209,706 (25.1)	223,622 (25.9)
Winter (Jun-Aug)	441,403 (26.0)	215,974 (25.9)	225,429 (26.2)
Prior admissions in 12 months			
0	711,166 (41.9)	411,233 (49.2)	300,379 (34.8)
1	374,576 (22.1)	186,092 (22.3)	188,760 (21.9)
≥2	610,370 (36.0)	238,009 (28.5)	372,928 (43.3)
Risk category based on admission unit			
1	659,004 (38.9)	324,467 (38.8)	334,991 (38.9)
2	503,703 (29.7)	245,034 (29.3)	259,133 (30.1)
3	387,474 (22.8)	192,039 (23.0)	195,701 (22.7)
4	145,931 (8.6)	73,794 (8.8)	72,242 (8.4)
In-hospital mortality (*n*, %)	35,287 (2.1)	17,535 (2.1)	17,752 (2.1)
Length of stay (days), (*n*, %)			
1–6	1284,508 (75.7)	629,370 (75.5)	655,138 (76.0)
7–13	248,154 (14.6)	122,166 (14.7)	125,988 (14.6)
14–20	83,037 (4.9)	41,317 (5.0)	41,720 (4.8)
21–27	35,457 (2.1)	17,696 (2.1)	17,761 (2.1)
≥28	44,956 (2.7)	23,496 (2.8)	21,460 (2.5)

DRG = diagnostic related group; ICU = intensive care unit.

**Table 2:** Details of bed utilization according to hospital length of stay and hospital type for patients hospitalized over two years in Victoria, Australia.

Days of Stay	Hospital Type	Number (%) Admissions *	Number (%) Bed Days
All length of stay	Overall	1,696,112 (100)	9,450,306 (100.0)
	Private	608,815 (35.9)	327,285 (34.6)
	Regional	207,938 (12.3)	1,148,083 (12.1)
	Secondary	382,490 (22.6)	1,984,477 (21.0)
	Tertiary	378,075 (22.3)	1,962,871 (20.8)
	Other	118,794 (7.0)	1,082,050 (11.4)
LOS1–6 days	Overall	1,284,508 (75.7)	2,988,383 (31.6)
	Private	450,324 (74.0)	1,047,298 (32.0)
	Regional	160,939 (77.4)	386,284 (33.6)
	Secondary	302,906 (79.2)	690,189 (34.8)
	Tertiary	294,359 (77.9)	678,895 (34.6)
	Other	75,980 (64.0)	185,717 (17.2)
LOS 7–13 days	Overall	248,154 (14.6)	2,285,159 (24.2)
	Private	102,152 (16.8)	945,255 (28.9)
	Regional	27,535 (13.2)	251,200 (21.9)
	Secondary	48,214 (12.6)	441,094 (22.2)
	Tertiary	51,832 (13.7)	475,350 (24.2)
	Other	18,421 (15.5)	172,260 (15.9)
LOS 14–20 days	Overall	83,037 (4.9)	1,354,911 (14.3)
	Private	32,485 (5.3)	524,364 (16.0)
	Regional	9446 (4.5)	155,169 (13.5)
	Secondary	15,066 (3.9)	247,272 (12.5)
	Tertiary	16,477 (4.4)	270,422 (13.8)
	Other	9563 (8.1)	157,684 (14.6)
LOS 21–27 days	Overall	35,457 (2.1)	831,620 (8.8)
	Private	11,866 (1.9)	2,77,342 (8.5)
	Regional	4251 (2.0)	99,921 (8.7)
	Secondary	6710 (1.8)	157,566 (7.9)
	Tertiary	7121 (1.9)	167,133 (8.5)
	Other	5509 (4.6)	129,658 (12.0)
LOS ≥ 28 days	Overall	44,956 (2.7)	1,990,233 (21.1)
	Private	11,988 (2.0)	478,566 (14.6)
	Regional	5767 (2.8)	255,509 (22.3)
	Secondary	9594 (2.5)	448,356 (22.6)
	Tertiary	8286 (2.2)	371,071 (18.9)
	Other	9321 (7.8)	436,731 (40.4)

* percentages are out of all stay for each group.

**Table 3:** Associations with prolonged hospitalization (≥14 days) and in-hospital mortality for patients hospitalized over two years in Victoria, Australia.

Variable	Longer LOS (Odds Ratio (OR), 95% CI)	Mortality (Hazard Ratio (HR) 95% CI)
Age groups, years		
20–24	0.74 (0.71–0.78)	0.22 (0.18–0.29)
25–29	0.85 (0.81–0.88)	0.26 (0.21–0.32)
30–34	0.97 (0.93–1.01)	0.32 (0.27–0.38)
35–39	1.01 (0.97–1.05)	0.40 (0.35–0.46)
40–44	0.97 (0.94–1.01)	0.56 (0.50–0.63)
45–49	0.95 (0.92–0.99)	0.70 (0.64–0.77)
50–54	0.96 (0.93–0.99)	0.80 (0.74–0.87)
55–59	0.95 (0.92–0.98)	0.91 (0.85–0.97)
60–64	1.0 [reference]	1.0 [reference]
65–69	1.03 (1.00–1.06)	1.08 (1.02–1.14)
70–74	1.16 (1.13–1.19)	1.18 (1.12–1.24)
75–79	1.47 (1.43–1.51)	1.32 (1.25–1.39)
80–84	1.80 (1.75–1.85)	1.62 (1.54–1.71)
≥85	2.32 (2.26–2.38)	2.50 (2.38–2.62)
Female gender	1.09 (1.08–1.11)	0.90 (0.88–0.92)
Indigenous population		
Yes	0.89 (0.83–0.95)	1.05 (0.90–1.21)
No	1.0 [reference]	1.0 [reference]
Undefined	1.13 (1.07–1.19)	1.34 (1.22–1.47)
Charlson co–morbidity Index		
0	1.0 [reference]	1.0 [reference]
1–2	1.44 (1.42–1.46)	2.74 (2.63–2.85)
3–4	2.04 (2.00–2.09)	4.27 (4.09–4.46)
5–6	2.15 (2.07–2.23)	6.15 (5.81–6.50)
>6	0.95 (0.93–0.98)	10.68 (10.24–11.13)
Marital Status		
Never married	1.59 (1.56–1.61)	0.89 (0.86–0.93)
Widowed	1.26 (1.23–1.28)	0.95 (0.92–0.97)
Married or defacto	1.0 [reference]	1.0 [reference]
Divorced or separated	1.37 (1.34–1.40)	0.86 (0.83–0.90)
Other	1.29 (1.24–1.35)	1.42 (1.32–1.52)
Current smoker	1.26 (1.24–1.28)	0.87 (0.83–0.91)
DRG type		
Medical	1.0 [reference]	1.0 [reference]
Surgical	0.60 (0.59–0.61)	0.28 (0.26–0.29)
Other	0.79 (0.76–0.82)	1.09 (1.04–1.14)
Emergency (unscheduled) admission	0.64 (0.63–0.65)	1.61 (1.56–1.65)
Admission source		
Home	1.0 [reference]	1.0 [reference]
Aged care residential facility	0.97 (0.90–1.04)	2.49 (2.33–2.67)
From another hospital	4.12 (4.06–4.18)	0.90 (0.87–0.93)
Other	4.24 (4.14–4.34)	1.80 (1.73–1.87)
ICU stay	3.38 (3.31–3.45)	3.17 (3.06–3.28)
Admitted on weekend	1.07 (1.05–1.08)	0.94 (0.91–0.96)
Season of admission		
Spring (Sep–Nov)	1.0 [reference]	1.0 [reference]
Summer (Dec–Feb)	1.17 (1.15–1.19)	0.96 (0.93–0.99)
Autumn (Mar–May)	1.15 (1.13–1.17)	0.98 (0.95–1.01)
Winter (Jun-Aug)	1.14 (1.13–1.16)	0.98 (0.95–1.01)
Prior admissions (in 12 months)		
0	1.0 [reference]	1.0 [reference]
1	1.22 (1.19–1.24)	0.85 (0.81–0.88)
≥2	1.32 (1.30–1.35)	0.98 (0.95–1.01)
Risk category based on admission unit		
1	1.0 [reference]	1.0 [reference]
2	1.86 (1.83–1.89)	0.91 (0.87–0.94)
3	2.42 (2.38–2.47)	1.38 (1.34–1.43)
4	8.39 (8.22–8.56)	2.59 (2.50–2.69)
Died in hospital	0.90 (0.87–0.93)	-

DRG = diagnostic related group, ICU = intensive care unit, LOS = length of stay. [reference], reference group.

## Supplementary Materials

The following are available online at https://www.mdpi.com/2077-0383/9/9/3055/s1, Table S1: Diagnostic related group (DRG) Codes for different specialties; Table S2: Uni-variate associations with prolonged hospital length of stay for patients hospitalized over two years in Victoria, Australia; Table S3: Associations with prolonged hospitalization (≥14 days) for patients hospitalized over two years in Victoria, Australia who were discharged alive; Table S4: Uni-variate associations with in-hospital mortality for patients hospitalized over two years in Victoria, Australia; Table S5: Nature and destination of discharge among people who survived in hospital for patients hospitalized over two years in Victoria, Australia; Table S6: Independent associations with hospital discharge to home for patients hospitalized over two years in Victoria, Australia; Table S7: Summary of literature describing epidemiology of prolonged hospitalizations 2015 to 2019.
