# Supplementary material for: The Frequency of, and Factors Associated with Prolonged Hospitalization: A Multicentre Study in Victoria, Australia
Source: J Clin Med. 2020 Sep 22;9(9):3055. doi: 10.3390/jcm9093055 (PMC7564707; doi:10.3390/jcm9093055)
Supplement: Supplementary file 1 [file jcm-09-03055-s001.pdf]

Supplementary materials

Table S1: Diagnostic related group (DRG) Codes for different specialties

Specialty	DRG Code	Description
Clinical Cardiology	F40A	Circulatory Disorders W Ventilator Support, Major Complexity
Clinical Cardiology	F40B	Circulatory Disorders W Ventilator Support, Minor Complexity
Clinical Cardiology	F42C	Circulatory Dsrds, Not Adm for AMI W Invasive Cardiac Inves, Sameday
Clinical Cardiology	F43A	Circulatory Disorders W Non-Invasive Ventilation, Major Complexity
Clinical Cardiology	F43B	Circulatory Disorders W Non-Invasive Ventilation, Minor Complexity
Clinical Cardiology	F43Z	Circulatory Disorders W Non-Invasive Ventilation
Clinical Cardiology	F60A	Circulatory Dsrd, Adm for AMI W/O Invas Card Inves Proc
Clinical Cardiology	F60B	Circulatory Dsrd, Adm for AMI W/O Invas Card Inves Proc, Transf <5 Days
Clinical Cardiology	F61A	Infective Endocarditis, Major Complexity
Clinical Cardiology	F61B	Infective Endocarditis, Minor Complexity
Clinical Cardiology	F62A	Heart Failure and Shock, Major Complexity
Clinical Cardiology	F62B	Heart Failure and Shock, Minor Complexity
Clinical Cardiology	F62C	Heart Failure and Shock, Transferred <5 Days
Clinical Cardiology	F63A	Venous Thrombosis, Major Complexity
Clinical Cardiology	F63B	Venous Thrombosis, Minor Complexity
Clinical Cardiology	F66A	Coronary Atherosclerosis, Major Complexity
Clinical Cardiology	F66B	Coronary Atherosclerosis, Minor Complexity
Clinical Cardiology	F67A	Hypertension, Major Complexity
Clinical Cardiology	F67B	Hypertension, Minor Complexity
Clinical Cardiology	F68A	Congenital Heart Disease, Major Complexity
Clinical Cardiology	F68B	Congenital Heart Disease, Minor Complexity
Clinical Cardiology	F68Z	Congenital Heart Disease
Clinical Cardiology	F69A	Valvular Disorders, Major Complexity
Clinical Cardiology	F69B	Valvular Disorders, Minor Complexity
Clinical Cardiology	F72A	Unstable Angina, Major Complexity
Clinical Cardiology	F72B	Unstable Angina, Minor Complexity
Clinical Cardiology	F74A	Chest Pain, Major Complexity
Clinical Cardiology	F74B	Chest Pain, Minor Complexity
Clinical Cardiology	F75A	Other Circulatory Disorders, Major Complexity
Clinical Cardiology	F75B	Other Circulatory Disorders, Intermediate Complexity
Clinical Cardiology	F75C	Other Circulatory Disorders, Minor Complexity
Clinical Cardiology	F76A	Arrhythmia, Cardiac Arrest and Conduction Disorders, Major Complexity
Clinical Cardiology	F76B	Arrhythmia, Cardiac Arrest and Conduction Disorders, Minor Complexity
Clinical Cardiology	F76C	Arrhythmia, Cardiac Arrest and Conduction Disorders, Sameday
Interventional Cardiology	F01A	Implantation and Replacement of AICD, Total System, Major Complexity
Interventional Cardiology	F01B	Implantation and Replacement of AICD, Total System, Minor Complexity
Interventional Cardiology	F02Z	Other AICD Procedures
Interventional Cardiology	F10A	Interventional Coronary Procedures, Admitted for AMI, Major Complexity
Interventional Cardiology	F10B	Interventional Coronary Procedures, Admitted for AMI, Minor Complexity
Interventional Cardiology	F12A	Implantation and Replacement of Pacemaker, Total System, Major Complexity
Interventional Cardiology	F12B	Implantation and Replacement of Pacemaker, Total System, Minor Complexity
Interventional Cardiology	F15A	Interventional Coronary Procs, Not Adm for AMI, W Stent Implant, Major Comp
Interventional Cardiology	F15B	Interventional Coronary Procs, Not Adm for AMI, W Stent Implant, Minor Comp

Interventional Cardiology	F16A	Interventional Coronary Procs, Not Adm for AMI, W/O Stent Implant, Major Comp
Interventional Cardiology	F16B	Interventional Coronary Procs, Not Adm for AMI, W/O Stent Implant, Minor Comp
Interventional Cardiology	F17A	Insertion and Replacement of Pacemaker Generator, Major Complexity
Interventional Cardiology	F17B	Insertion and Replacement of Pacemaker Generator, Minor Complexity
Interventional Cardiology	F17Z	Insertion or Replacement of Pacemaker Generator
Interventional Cardiology	F18A	Other Pacemaker Procedures, Major Complexity
Interventional Cardiology	F18B	Other Pacemaker Procedures, Minor Complexity
Interventional Cardiology	F19A	Trans-Vascular Percutaneous Cardiac Intervention, Major Complexity
Interventional Cardiology	F19B	Trans-Vascular Percutaneous Cardiac Intervention, Minor Complexity
Interventional Cardiology	F41A	Circulatory Disorders, Adm for AMI W Invasive Cardiac Inves Proc, Major Comp
Interventional Cardiology	F41B	Circulatory Disorders, Adm for AMI W Invasive Cardiac Inves Proc, Minor Comp
Interventional Cardiology	F42A	Circulatory Dsrds, Not Adm for AMI W Invasive Cardiac Inves Proc, Major Comp
Interventional Cardiology	F42B	Circulatory Dsrds, Not Adm for AMI W Invasive Cardiac Inves Proc, Minor Comp
Dermatology	J67A	Minor Skin Disorders, Major Complexity
Dermatology	J67B	Minor Skin Disorders, Minor Complexity
Dermatology	J68A	Major Skin Disorders, Major Complexity
Dermatology	J68B	Major Skin Disorders, Minor Complexity
Dermatology	J68C	Major Skin Disorders, Sameday
Dermatology	K09C	Other Endocrine, Nutritional and Metabolic OR Procs W/O CC
Endocrinology	K60A	Diabetes, Major Complexity
Endocrinology	K60B	Diabetes, Minor Complexity
Endocrinology	K60C	Diabetes, Sameday
Endocrinology	K61Z	Severe Nutritional Disturbance
Endocrinology	K62A	Miscellaneous Metabolic Disorders, Major Complexity
Endocrinology	K62B	Miscellaneous Metabolic Disorders, Intermediate Complexity
Endocrinology	K62C	Miscellaneous Metabolic Disorders, Minor Complexity
Endocrinology	K63A	Inborn Errors of Metabolism, Major Complexity
Endocrinology	K63B	Inborn Errors of Metabolism, Minor Complexity
Endocrinology	K64A	Endocrine Disorders, Major Complexity
Endocrinology	K64B	Endocrine Disorders, Minor Complexity
Endocrinology	K64C	Endocrine Disorders, Sameday
Gastroenterology	G61A	Gastrointestinal Haemorrhage, Major Complexity
Gastroenterology	G61B	Gastrointestinal Haemorrhage, Minor Complexity
Gastroenterology	G64A	Inflammatory Bowel Disease, Major Complexity
Gastroenterology	G64B	Inflammatory Bowel Disease, Minor Complexity
Gastroenterology	G67A	Oesophagitis and Gastroenteritis, Major Complexity
Gastroenterology	G67B	Oesophagitis and Gastroenteritis, Minor Complexity
Gastroenterology	H40A	Endoscopic Procedures for Bleeding Oesophageal Varices, Major Complexity
Gastroenterology	H40B	Endoscopic Procedures for Bleeding Oesophageal Varices, Intermediate Complexity
Gastroenterology	H40C	Endoscopic Procedures for Bleeding Oesophageal Varices, Minor Complexity
Gastroenterology	H60A	Cirrhosis and Alcoholic Hepatitis, Major Complexity
Gastroenterology	H60B	Cirrhosis and Alcoholic Hepatitis, Intermediate Complexity
Gastroenterology	H60C	Cirrhosis and Alcoholic Hepatitis, Minor Complexity
Gastroenterology	H63A	Other Disorders of Liver, Major Complexity

Gastroenterology	H63B	Other Disorders of Liver, Intermediate Complexity
Gastroenterology	H63C	Other Disorders of Liver, Minor Complexity
Gastroenterology	K40A	Endoscopic and Investigative Procedures for Metabolic Disorders, Major Comp
Gastroenterology	K40B	Endoscopic and Investigative Procedures for Metabolic Disorders, Minor Comp
Gastroenterology	K40C	Endoscopic and Investigative Procs for Metabolic Disorders, Sameday
Gastroenterology	K61A	Severe Nutritional Disturbance, Major Complexity
Gastroenterology	K61B	Severe Nutritional Disturbance, Minor Complexity
Diagnostic GI Endoscopy	G46A	Complex Endoscopy, Major Complexity
Diagnostic GI Endoscopy	G46B	Complex Endoscopy, Minor Complexity
Diagnostic GI Endoscopy	G46C	Complex Endoscopy, Sameday
Diagnostic GI Endoscopy	G47A	Gastroscopy, Major Complexity
Diagnostic GI Endoscopy	G47B	Gastroscopy, Intermediate Complexity
Diagnostic GI Endoscopy	G47C	Gastroscopy, Minor Complexity
Diagnostic GI Endoscopy	G48A	Colonoscopy, Major Complexity
Diagnostic GI Endoscopy	G48B	Colonoscopy, Minor Complexity
Diagnostic GI Endoscopy	G48C	Colonoscopy, Sameday
Diagnostic GI Endoscopy	H43A	ERCP Procedures, Major Complexity
Diagnostic GI Endoscopy	H43B	ERCP Procedures, Intermediate Complexity
Diagnostic GI Endoscopy	H43C	ERCP Procedures, Minor Complexity
Haematology	B40Z	Plasmapheresis W Neurological Disease, Sameday
Haematology	B62Z	Apheresis
Haematology	Q61A	Red Blood Cell Disorders, Major Complexity
Haematology	Q61B	Red Blood Cell Disorders, Intermediate Complexity
Haematology	Q61C	Red Blood Cell Disorders, Minor Complexity
Haematology	Q62A	Coagulation Disorders, Major Complexity
Haematology	Q62B	Coagulation Disorders, Minor Complexity
Haematology	R01A	Lymphoma and Leukaemia W Major OR Procedures, Major Complexity
Haematology	R01B	Lymphoma and Leukaemia W Major OR Procedures, Minor Complexity
Haematology	R03A	Lymphoma and Leukaemia W Other OR Procedures, Major Complexity
Haematology	R03B	Lymphoma and Leukaemia W Other OR Procedures, Intermediate Complexity
Haematology	R03C	Lymphoma and Leukaemia W Other OR Procedures, Minor Complexity
Haematology	R60A	Acute Leukaemia, Major Complexity
Haematology	R60B	Acute Leukaemia, Minor Complexity
Haematology	R60C	Acute Leukaemia, Sameday
Haematology	R61A	Lymphoma and Non-Acute Leukaemia, Major Complexity
Haematology	R61B	Lymphoma and Non-Acute Leukaemia, Minor Complexity
Haematology	R61C	Lymphoma and Non-Acute Leukaemia, Sameday
Haematology	J64A	Cellulitis, Major Complexity
Immunology & Infections	J64B	Cellulitis, Minor Complexity
Immunology & Infections	Q60A	Reticuloendothelial and Immunity Disorders, Major Complexity
Immunology & Infections	Q60B	Reticuloendothelial and Immunity Disorders, Minor Complexity
Immunology & Infections	Q60C	Reticuloendothelial and Immunity Disorders, Sameday
Immunology & Infections	S65A	Human Immunodeficiency Virus, Major Complexity
Immunology & Infections	S65B	Human Immunodeficiency Virus, Intermediate Complexity
Immunology & Infections	S65C	Human Immunodeficiency Virus, Minor Complexity
Immunology & Infections	S65D	Human Immunodeficiency Virus, Sameday
Immunology & Infections	T40Z	Infectious and Parasitic Diseases W Ventilator Support
Immunology & Infections	T60A	Septicaemia, Major Complexity
Immunology & Infections	T60B	Septicaemia, Intermediate Complexity

Immunology & Infections	T60C	Septicaemia, Minor Complexity
Immunology & Infections	T62A	Fever of Unknown Origin, Major Complexity
Immunology & Infections	T62B	Fever of Unknown Origin, Minor Complexity
Immunology & Infections	T64A	Other Infectious and Parasitic Diseases, Major Complexity
Immunology & Infections	T64B	Other Infectious and Parasitic Diseases, Intermediate Complexity
Immunology & Infections	T64C	Other Infectious and Parasitic Diseases, Minor Complexity
Oncology	B66A	Nervous System Neoplasms, Major Complexity
Oncology	B66B	Nervous System Neoplasms, Minor Complexity
Oncology	B66C	Nervous System Neoplasms W/O Radiotherapy W/O Catastrophic or Severe CC
Oncology	D02B	Head and Neck Procedures, Intermediate Complexity
Oncology	D60A	Ear, Nose, Mouth and Throat Malignancy, Major Complexity
Oncology	D60B	Ear, Nose, Mouth and Throat Malignancy, Minor Complexity
Oncology	D60C	Ear, Nose, Mouth and Throat Malignancy, Sameday
Oncology	E71A	Respiratory Neoplasms, Major Complexity
Oncology	E71B	Respiratory Neoplasms, Minor Complexity
Oncology	E71C	Respiratory Neoplasms, Sameday
Oncology	G03A	Stomach, Oesophageal and Duodenal Procedures, Major Complexity
Oncology	G07A	Appendicectomy, Major Complexity
Oncology	G60A	Digestive Malignancy, Major Complexity
Oncology	G60B	Digestive Malignancy, Minor Complexity
Oncology	H61A	Malignancy of Hepatobiliary System and Pancreas, Major Complexity
Oncology	H61B	Malignancy of Hepatobiliary System and Pancreas, Minor Complexity
Oncology	H61C	Malignancy of Hepatobiliary System and Pancreas, Sameday
Oncology	I65A	Musculoskeletal Malignant Neoplasms, Major Complexity
Oncology	I65B	Musculoskeletal Malignant Neoplasms, Minor Complexity
Oncology	J62A	Malignant Breast Disorders, Major Complexity
Oncology	J62B	Malignant Breast Disorders, Minor Complexity
Oncology	J69A	Skin Malignancy, Major Complexity
Oncology	J69B	Skin Malignancy, Intermediate Complexity
Oncology	J69C	Skin Malignancy, Minor Complexity
Oncology	L03A	Kidney, Ureter and Major Bladder Procedures for Neoplasm, Major Complexity
Oncology	L03B	Kidney, Ureter and Major Bladder Procedures for Neoplasm, Intermediate Comp
Oncology	L03C	Kidney, Ureter and Major Bladder Procedures for Neoplasm, Minor Complexity
Oncology	L62A	Kidney and Urinary Tract Neoplasms, Major Complexity
Oncology	L62B	Kidney and Urinary Tract Neoplasms, Minor Complexity
Oncology	M60A	Male Reproductive System Malignancy, Major Complexity
Oncology	M60B	Male Reproductive System Malignancy, Minor Complexity
Oncology	N12A	Uterus and Adnexa Procedures for Malignancy, Major Complexity
Oncology	N12B	Uterus and Adnexa Procedures for Malignancy, Intermediate Complexity
Oncology	N12C	Uterus and Adnexa Procedures for Malignancy, Minor Complexity
Oncology	N60A	Female Reproductive System Malignancy, Major Complexity
Oncology	N60B	Female Reproductive System Malignancy, Minor Complexity
Oncology	R02A	Other Neoplastic Disorders W Major OR Procedures, Major Complexity
Oncology	R02B	Other Neoplastic Disorders W Major OR Procedures, Intermediate Complexity
Oncology	R02C	Other Neoplastic Disorders W Major OR Procedures, Minor Complexity
Oncology	R04A	Other Neoplastic Disorders W Other OR Procedures, Major Complexity
Oncology	R04B	Other Neoplastic Disorders W Other OR Procedures, Minor Complexity

Oncology	R62A	Other Neoplastic Disorders, Major Complexity
Oncology	R62B	Other Neoplastic Disorders, Intermediate Complexity
Oncology	R62C	Other Neoplastic Disorders, Minor Complexity
Chemotherapy & Radiotherapy	R63Z	Chemotherapy
Chemotherapy & Radiotherapy	R64Z	Radiotherapy
Neurology	B41Z	Telemetric EEG Monitoring
Neurology	B42A	Nervous System Disorders W Ventilator Support, Major Complexity
Neurology	B42B	Nervous System Disorders W Ventilator Support, Minor Complexity
Neurology	B60A	Acute Paraplegia and Quadriplegia W or W/O OR Procedures, Major Complexity
Neurology	B60B	Acute Paraplegia and Quadriplegia W or W/O OR Procedures, Minor Complexity
Neurology	B65A	Cerebral Palsy, Major Complexity
Neurology	B65B	Cerebral Palsy, Minor Complexity
Neurology	B67A	Degenerative Nervous System Disorders, Major Complexity
Neurology	B67B	Degenerative Nervous System Disorders, Intermediate Complexity
Neurology	B67C	Degenerative Nervous System Disorders, Minor Complexity
Neurology	B68A	Multiple Sclerosis and Cerebellar Ataxia, Major Complexity
Neurology	B68B	Multiple Sclerosis and Cerebellar Ataxia, Minor Complexity
Neurology	B69A	TIA and Precerebral Occlusion, Major Complexity
Neurology	B69B	TIA and Precerebral Occlusion, Minor Complexity
Neurology	B70A	Stroke and Other Cerebrovascular Disorders, Major Complexity
Neurology	B70B	Stroke and Other Cerebrovascular Disorders, Intermediate Complexity
Neurology	B70C	Stroke and Other Cerebrovascular Disorders, Minor Complexity
Neurology	B70D	Stroke and Other Cerebrovascular Disorders, Transferred <5 Days
Neurology	B71A	Cranial and Peripheral Nerve Disorders, Major Complexity
Neurology	B71B	Cranial and Peripheral Nerve Disorders, Minor Complexity
Neurology	B71C	Cranial and Peripheral Nerve Disorders, Sameday
Neurology	B72A	Nervous System Infection Except Viral Meningitis, Major Complexity
Neurology	B72B	Nervous System Infection Except Viral Meningitis, Minor Complexity
Neurology	B73Z	Viral Meningitis
Neurology	B76A	Seizures, Major Complexity
Neurology	B76B	Seizures, Minor Complexity
Neurology	B76C	Seizures, Sameday
Neurology	B77A	Headaches, Major Complexity
Neurology	B77B	Headaches, Minor Complexity
Neurology	B77Z	Headache
Neurology	B81A	Other Disorders of the Nervous System, Major Complexity
Neurology	B81B	Other Disorders of the Nervous System, Minor Complexity
Neurology	B82A	Chronic & Unspec Para/Quadriplegia W or W/O OR Proc, Major Complexity
Neurology	B82B	Chronic & Unspec Para/Quadriplegia W or W/O OR Proc, Intermediate Complexity
Neurology	B82C	Chronic & Unspec Para/Quadriplegia W or W/O OR Proc, Minor Complexity
Neurology	C61A	Neurological and Vascular Disorders of the Eye, Major Complexity
Neurology	C61B	Neurological and Vascular Disorders of the Eye, Minor Complexity
Neurology	D61A	Dysequilibrium, Major Complexity
Neurology	D61B	Dysequilibrium, Minor Complexity
Neurology	D61C	Dysequilibrium, Sameday
Renal Medicine	L02A	Operative Insertion of Peritoneal Catheter for Dialysis, Major Complexity

Renal Medicine	L02B	Operative Insertion of Peritoneal Catheter for Dialysis, Minor Complexity
Renal Medicine	L09A	Other Procedures for Kidney and Urinary Tract Disorders, Major Complexity
Renal Medicine	L09B	Other Procedures for Kidney and Urinary Tract Disorders, Intermediate Complexity
Renal Medicine	L09C	Other Procedures for Kidney and Urinary Tract Disorders, Minor Complexity
Renal Medicine	L60A	Kidney Failure, Major Complexity
Renal Medicine	L60B	Kidney Failure, Intermediate Complexity
Renal Medicine	L60C	Kidney Failure, Minor Complexity
Renal Medicine	L67A	Other Kidney and Urinary Tract Disorders, Major Complexity
Renal Medicine	L67B	Other Kidney and Urinary Tract Disorders, Intermediate Complexity
Renal Medicine	L67C	Other Kidney and Urinary Tract Disorders, Minor Complexity
Respiratory Medicine	A40A	ECMO, Major Complexity
Respiratory Medicine	A40B	ECMO, Minor Complexity
Respiratory Medicine	D66C	Other Ear, Nose, Mouth and Throat Disorders, Sameday
Respiratory Medicine	E02A	Other Respiratory System OR Procedures, Major Complexity
Respiratory Medicine	E02B	Other Respiratory System OR Procedures, Intermediate Complexity
Respiratory Medicine	E02C	Other Respiratory System OR Procedures, Minor Complexity
Respiratory Medicine	E40A	Respiratory System Disorders W Ventilator Support, Major Complexity
Respiratory Medicine	E40B	Respiratory System Disorders W Ventilator Support, Minor Complexity
Respiratory Medicine	E41A	Respiratory System Disorders W Non-Invasive Ventilation, Major Complexity
Respiratory Medicine	E41B	Respiratory System Disorders W Non-Invasive Ventilation, Minor Complexity
Respiratory Medicine	E42A	Bronchoscopy, Major Complexity
Respiratory Medicine	E42B	Bronchoscopy, Minor Complexity
Respiratory Medicine	E42C	Bronchoscopy, Sameday
Respiratory Medicine	E60A	Cystic Fibrosis, Major Complexity
Respiratory Medicine	E60B	Cystic Fibrosis, Minor Complexity
Respiratory Medicine	E61A	Pulmonary Embolism, Major Complexity
Respiratory Medicine	E61B	Pulmonary Embolism, Minor Complexity
Respiratory Medicine	E62A	Respiratory Infections and Inflammations, Major Complexity
Respiratory Medicine	E62B	Respiratory Infections and Inflammations, Minor Complexity
Respiratory Medicine	E62C	Respiratory Infections/Inflammations W/O CC
Respiratory Medicine	E63A	Sleep Apnoea, Major Complexity
Respiratory Medicine	E63B	Sleep Apnoea, Minor Complexity
Respiratory Medicine	E63Z	Sleep Apnoea
Respiratory Medicine	E64A	Pulmonary Oedema and Respiratory Failure, Major Complexity
Respiratory Medicine	E64B	Pulmonary Oedema and Respiratory Failure, Minor Complexity
Respiratory Medicine	E65A	Chronic Obstructive Airways Disease, Major Complexity
Respiratory Medicine	E65B	Chronic Obstructive Airways Disease, Minor Complexity
Respiratory Medicine	E67A	Respiratory Signs and Symptoms, Major Complexity
Respiratory Medicine	E67B	Respiratory Signs and Symptoms, Minor Complexity
Respiratory Medicine	E68A	Pneumothorax, Major Complexity
Respiratory Medicine	E68B	Pneumothorax, Minor Complexity
Respiratory Medicine	E69A	Bronchitis and Asthma, Major Complexity
Respiratory Medicine	E69B	Bronchitis and Asthma, Minor Complexity
Respiratory Medicine	E72Z	Respiratory Problems Arising from Neonatal Period
Respiratory Medicine	E73A	Pleural Effusion, Major Complexity
Respiratory Medicine	E73B	Pleural Effusion, Intermediate Complexity
Respiratory Medicine	E73C	Pleural Effusion, Minor Complexity

Respiratory Medicine	E74A	Interstitial Lung Disease, Major Complexity
Respiratory Medicine	E74B	Interstitial Lung Disease, Minor Complexity
Respiratory Medicine	E74C	Interstitial Lung Disease W/O CC
Respiratory Medicine	E75A	Other Respiratory System Disorders, Major Complexity
Respiratory Medicine	E75B	Other Respiratory System Disorders, Minor Complexity
Respiratory Medicine	E76A	Respiratory Tuberculosis, Major Complexity
Respiratory Medicine	E76B	Respiratory Tuberculosis, Minor Complexity
Rheumatology	I66A	Inflammatory Musculoskeletal Disorders, Major Complexity
Rheumatology	I66B	Inflammatory Musculoskeletal Disorders, Intermediate Complexity
Rheumatology	I66C	Inflammatory Musculoskeletal Disorders, Minor Complexity
Rheumatology	I71A	Other Musculotendinous Disorders, Major Complexity
Rheumatology	I71B	Other Musculotendinous Disorders, Minor Complexity
Rheumatology	I72A	Specific Musculotendinous Disorders, Major Complexity
Rheumatology	I72B	Specific Musculotendinous Disorders, Minor Complexity
Non Subspecialty Medicine	B63A	Dementia and Other Chronic Disturbances of Cerebral Function, Major Complexity
Non Subspecialty Medicine	B63B	Dementia and Other Chronic Disturbances of Cerebral Function, Minor Complexity
Non Subspecialty Medicine	B63Z	Dementia and Other Chronic Disturbances of Cerebral Function
Non Subspecialty Medicine	B64A	Delirium, Major Complexity
Non Subspecialty Medicine	B64B	Delirium, Minor Complexity
Non Subspecialty Medicine	B74A	Nontraumatic Stupor and Coma, Major Complexity
Non Subspecialty Medicine	B74B	Nontraumatic Stupor and Coma, Minor Complexity
Non Subspecialty Medicine	B75Z	Febrile Convulsions
Non Subspecialty Medicine	D63C	Otitis Media and Upper Respiratory Infections, Sameday
Non Subspecialty Medicine	D64A	Laryngotracheitis and Epiglottitis, Major Complexity
Non Subspecialty Medicine	D64B	Laryngotracheitis and Epiglottitis, Minor Complexity
Non Subspecialty Medicine	D64Z	Laryngotracheitis and Epiglottitis
Non Subspecialty Medicine	D65A	Nasal Trauma and Deformity, Major Complexity
Non Subspecialty Medicine	D65B	Nasal Trauma and Deformity, Minor Complexity
Non Subspecialty Medicine	D65Z	Nasal Trauma and Deformity
Non Subspecialty Medicine	E70A	Whooping Cough and Acute Bronchiolitis, Major Complexity
Non Subspecialty Medicine	E70B	Whooping Cough and Acute Bronchiolitis, Minor Complexity
Non Subspecialty Medicine	F73A	Syncope and Collapse, Major Complexity
Non Subspecialty Medicine	F73B	Syncope and Collapse, Minor Complexity
Non Subspecialty Medicine	F73C	Syncope and Collapse, Sameday
Non Subspecialty Medicine	I40Z	Infusions for Musculoskeletal Disorders, Sameday
Non Subspecialty Medicine	L63A	Kidney and Urinary Tract Infections, Major Complexity
Non Subspecialty Medicine	L63B	Kidney and Urinary Tract Infections, Minor Complexity
Non Subspecialty Medicine	T63A	Viral Illnesses, Major Complexity
Non Subspecialty Medicine	T63B	Viral Illnesses, Minor Complexity
Non Subspecialty Medicine	X61A	Allergic Reactions, Major Complexity
Non Subspecialty Medicine	X61B	Allergic Reactions, Minor Complexity
Non Subspecialty Medicine	X61Z	Allergic Reactions
Non Subspecialty Medicine	X64A	Other Injuries, Poisonings and Toxic Effects, Major Complexity
Non Subspecialty Medicine	X64B	Other Injuries, Poisonings and Toxic Effects, Minor Complexity
Non Subspecialty Medicine	Z40Z	Other Contacts W Health Services W Endoscopy, Sameday
Non Subspecialty Medicine	Z61A	Signs and Symptoms, Major Complexity
Non Subspecialty Medicine	Z61B	Signs and Symptoms, Intermediate Complexity
Non Subspecialty Medicine	Z61C	Signs and Symptoms, Minor Complexity
Non Subspecialty Medicine	Z63A	Other Follow Up After Surgery or Medical Care, Major Complexity
Non Subspecialty Medicine	Z63B	Other Follow Up After Surgery or Medical Care, Minor Complexity

Non Subspecialty Medicine	Z64A	Other Factors Influencing Health Status, Major Complexity
Non Subspecialty Medicine	Z64B	Other Factors Influencing Health Status, Minor Complexity
Non Subspecialty Medicine	Z65Z	Congenital Anomalies and Problems Arising from Neonatal Period
Non Subspecialty Medicine	Z66Z	Sleep Disorders
Breast Surgery	J06A	Major Procedures for Breast Disorders, Major Complexity
Breast Surgery	J06B	Major Procedures for Breast Disorders, Minor Complexity
Breast Surgery	J07A	Minor Procedures for Breast Disorders, Major Complexity
Breast Surgery	J07B	Minor Procedures for Breast Disorders, Minor Complexity
Breast Surgery	J14Z	Major Breast Reconstructions
Breast Surgery	J63A	Non-Malignant Breast Disorders, Major Complexity
Breast Surgery	J63B	Non-Malignant Breast Disorders, Minor Complexity
Cardiothoracic Surgery	A10Z	Insertion of Ventricular Assist Device
Cardiothoracic Surgery	E01A	Major Chest Procedures, Major Complexity
Cardiothoracic Surgery	E01B	Major Chest Procedures, Intermediate Complexity
Cardiothoracic Surgery	E01C	Major Chest Procedures, Minor Complexity
Cardiothoracic Surgery	F03A	Cardiac Valve Procedures W CPB Pump W Invasive Cardiac Investigation, Major Comp
Cardiothoracic Surgery	F03B	Cardiac Valve Procedures W CPB Pump W Invasive Cardiac Investigation, Minor Comp
Cardiothoracic Surgery	F04A	Cardiac Valve Procedures W CPB Pump W/O Invasive Cardiac Invest, Major Comp
Cardiothoracic Surgery	F04B	Cardiac Valve Procedures W CPB Pump W/O Invasive Cardiac Invest, Interm Comp
Cardiothoracic Surgery	F04C	Cardiac Valve Procedures W CPB Pump W/O Invasive Cardiac Invest, Minor Comp
Cardiothoracic Surgery	F05A	Coronary Bypass W Invasive Cardiac Investigation, Major Complexity
Cardiothoracic Surgery	F05B	Coronary Bypass W Invasive Cardiac Investigation, Minor Complexity
Cardiothoracic Surgery	F06A	Coronary Bypass W/O Invasive Cardiac Investigation, Major Complexity
Cardiothoracic Surgery	F06B	Coronary Bypass W/O Invasive Cardiac Investigation, Minor Complexity
Cardiothoracic Surgery	F07A	Other Cardiothoracic/Vascular Procedures W CPB Pump, Major Complexity
Cardiothoracic Surgery	F07B	Other Cardiothoracic/Vascular Procedures W CPB Pump, Intermediate Complexity
Cardiothoracic Surgery	F07C	Other Cardiothoracic/Vascular Procedures W CPB Pump, Minor Complexity
Cardiothoracic Surgery	F09A	Other Cardiothoracic Procedures W/O CPB Pump, Major Complexity
Cardiothoracic Surgery	F09B	Other Cardiothoracic Procedures W/O CPB Pump, Intermediate Complexity
Cardiothoracic Surgery	F09C	Other Cardiothoracic Procedures W/O CPB Pump, Minor Complexity
Cardiothoracic Surgery	P02Z	Cardiothoracic and Vascular Procedures for Neonates
Colorectal Surgery	G01A	Rectal Resection, Major Complexity
Colorectal Surgery	G01B	Rectal Resection, Intermediate Complexity
Colorectal Surgery	G01C	Rectal Resection, Minor Complexity
Colorectal Surgery	G02A	Major Small and Large Bowel Procedures, Major Complexity
Colorectal Surgery	G02B	Major Small and Large Bowel Procedures, Intermediate Complexity
Colorectal Surgery	G02C	Major Small and Large Bowel Procedures, Minor Complexity
Colorectal Surgery	G05A	Minor Small and Large Bowel Procedures, Major Complexity
Colorectal Surgery	G05B	Minor Small and Large Bowel Procedures, Minor Complexity
Colorectal Surgery	G05C	Minor Small and Large Bowel Procedures W/O CC
Colorectal Surgery	G11A	Anal and Stomal Procedures, Major Complexity
Colorectal Surgery	G11B	Anal and Stomal Procedures, Minor Complexity
Colorectal Surgery	G11Z	Anal and Stomal Procedures
Colorectal Surgery	J09Z	Perianal and Pilonidal Procedures

Upper GIT Surgery	G03B	Stomach, Oesophageal and Duodenal Procedures, Intermediate Complexity
Upper GIT Surgery	G03C	Stomach, Oesophageal and Duodenal Procedures, Minor Complexity
Upper GIT Surgery	G06Z	Pyloromyotomy
Upper GIT Surgery	H01A	Pancreas, Liver and Shunt Procedures, Major Complexity
Upper GIT Surgery	H01B	Pancreas, Liver and Shunt Procedures, Intermediate Complexity
Upper GIT Surgery	H01C	Pancreas, Liver and Shunt Procedures, Minor Complexity
Upper GIT Surgery	H02A	Major Biliary Tract Procedures, Major Complexity
Upper GIT Surgery	H02B	Major Biliary Tract Procedures, Minor Complexity
Upper GIT Surgery	H05A	Hepatobiliary Diagnostic Procedures, Major Complexity
Upper GIT Surgery	H05B	Hepatobiliary Diagnostic Procedures, Minor Complexity
Upper GIT Surgery	H06A	Other Hepatobiliary and Pancreas OR Procedures, Major Complexity
Upper GIT Surgery	H06B	Other Hepatobiliary and Pancreas OR Procedures, Intermediate Complexity
Upper GIT Surgery	H06C	Other Hepatobiliary and Pancreas OR Procedures, Minor Complexity
Upper GIT Surgery	H07A	Open Cholecystectomy, Major Complexity
Upper GIT Surgery	H07B	Open Cholecystectomy, Intermediate Complexity
Upper GIT Surgery	H07C	Open Cholecystectomy, Minor Complexity
Upper GIT Surgery	H08A	Laparoscopic Cholecystectomy, Major Complexity
Upper GIT Surgery	H08B	Laparoscopic Cholecystectomy, Minor Complexity
Upper GIT Surgery	H62A	Disorders of Pancreas, Except Malignancy, Major Complexity
Upper GIT Surgery	H62B	Disorders of Pancreas, Except Malignancy, Minor Complexity
Upper GIT Surgery	H64A	Disorders of the Biliary Tract, Major Complexity
Upper GIT Surgery	H64B	Disorders of the Biliary Tract, Minor Complexity
Upper GIT Surgery	H64C	Disorders of the Biliary Tract, Sameday
Upper GIT Surgery	K10A	Revisional and Open Bariatric Procedures, Major Complexity
Upper GIT Surgery	K10B	Revisional and Open Bariatric Procedures, Minor Complexity
Upper GIT Surgery	K11A	Major Laparoscopic Bariatric Procedures, Major Complexity
Upper GIT Surgery	K11B	Major Laparoscopic Bariatric Procedures, Minor Complexity
Upper GIT Surgery	K12A	Other Bariatric Procedures, Major Complexity
Upper GIT Surgery	K12B	Other Bariatric Procedures, Minor Complexity
Upper GIT Surgery	K12Z	Other Bariatric Procedures
Upper GIT Surgery	Q01A	Splenectomy, Major Complexity
Upper GIT Surgery	Q01B	Splenectomy, Minor Complexity
Upper GIT Surgery	W03Z	Abdominal Procedures for Multiple Significant Trauma
Head & Neck Surgery	D02A	Head and Neck Procedures, Major Complexity
Head & Neck Surgery	D02C	Head and Neck Procedures, Minor Complexity
Head & Neck Surgery	D05Z	Parotid Gland Procedures
Head & Neck Surgery	D14A	Mouth and Salivary Gland Procedures, Major Complexity
Head & Neck Surgery	D14B	Mouth and Salivary Gland Procedures, Minor Complexity
Head & Neck Surgery	K05A	Parathyroid Procedures, Major Complexity
Head & Neck Surgery	K05B	Parathyroid Procedures, Minor Complexity
Head & Neck Surgery	K06A	Thyroid Procedures, Major Complexity
Head & Neck Surgery	K06B	Thyroid Procedures, Minor Complexity
Head & Neck Surgery	K08Z	Thyroglossal Procedures
Neurosurgery	A12Z	Insertion of Neurostimulator Device
Neurosurgery	B01A	Ventricular Shunt Revision, Major Complexity
Neurosurgery	B01B	Ventricular Shunt Revision, Minor Complexity
Neurosurgery	B02A	Cranial Procedures, Major Complexity
Neurosurgery	B02B	Cranial Procedures, Intermediate Complexity
Neurosurgery	B02C	Cranial Procedures, Minor Complexity
Neurosurgery	B02Y	Endovascular clot retrieval

Neurosurgery	B03A	Spinal Procedures, Major Complexity
Neurosurgery	B03B	Spinal Procedures, Intermediate Complexity
Neurosurgery	B03C	Spinal Procedures, Minor Complexity
Neurosurgery	B06A	Procedures for Cerebral Palsy, Muscular Dystrophy and Neuropathy, Major Comp
Neurosurgery	B06B	Procedures for Cerebral Palsy, Muscular Dystrophy and Neuropathy, Interm Comp
Neurosurgery	B06C	Procedures for Cerebral Palsy, Muscular Dystrophy and Neuropathy, Minor Comp
Neurosurgery	B61A	Spinal Cord Conditions W or W/O OR Procedures, Major Complexity
Neurosurgery	B61B	Spinal Cord Conditions W or W/O OR Procedures, Minor Complexity
Neurosurgery	B78A	Intracranial Injuries, Major Complexity
Neurosurgery	B78B	Intracranial Injuries, Minor Complexity
Neurosurgery	B78C	Intracranial Injuries, Transferred <5 Days
Neurosurgery	B79A	Skull Fractures, Major Complexity
Neurosurgery	B79B	Skull Fractures, Minor Complexity
Neurosurgery	B80A	Other Head Injuries, Major Complexity
Neurosurgery	B80B	Other Head Injuries, Minor Complexity
Neurosurgery	I09A	Spinal Fusion, Major Complexity
Neurosurgery	I09B	Spinal Fusion, Intermediate Complexity
Neurosurgery	I09C	Spinal Fusion, Minor Complexity
Neurosurgery	I10A	Other Back and Neck Procedures, Major Complexity
Neurosurgery	I10B	Other Back and Neck Procedures, Minor Complexity
Neurosurgery	I15A	Cranio-Facial Surgery, Major Complexity
Neurosurgery	I15B	Cranio-Facial Surgery, Minor Complexity
Neurosurgery	I15Z	Cranio-Facial Surgery
Neurosurgery	K02A	Pituitary Procedures, Major Complexity
Neurosurgery	K02B	Pituitary Procedures, Minor Complexity
Neurosurgery	W01A	Vent, Trac & Cran Procs for Mult Sig Trauma, Major Complexity
Neurosurgery	W01B	Vent, Trac & Cran Procs for Mult Sig Trauma, Intermediate Complexity
Neurosurgery	W01C	Vent, Trac & Cran Procs for Mult Sig Trauma, Minor Complexity
Neurosurgery	W60A	Multiple Sig Trauma, Died or Transferred to Acute Facility <5 Days, Major Comp
Neurosurgery	W60B	Multiple Sig Trauma, Died or Transferred to Acute Facility <5 Days, Minor Comp
Dentistry	D40Z	Dental Extractions and Restorations
Ear, Nose & Throat	D01Z	Cochlear Implant
Ear, Nose & Throat	D06Z	Sinus and Complex Middle Ear Procedures
Ear, Nose & Throat	D10Z	Nasal Procedures
Ear, Nose & Throat	D11Z	Tonsillectomy and Adenoidectomy
Ear, Nose & Throat	D12A	Other Ear, Nose, Mouth and Throat Procedures, Major Complexity
Ear, Nose & Throat	D12B	Other Ear, Nose, Mouth and Throat Procedures, Minor Complexity
Ear, Nose & Throat	D13Z	Myringotomy W Tube Insertion
Ear, Nose & Throat	D15Z	Mastoid Procedures
Ear, Nose & Throat	D62A	Epistaxis, Major Complexity
Ear, Nose & Throat	D62B	Epistaxis, Minor Complexity
Ear, Nose & Throat	D63A	Otitis Media and Upper Respiratory Infections, Major Complexity
Ear, Nose & Throat	D63B	Otitis Media and Upper Respiratory Infections, Minor Complexity
Ear, Nose & Throat	D66A	Other Ear, Nose, Mouth and Throat Disorders, Major Complexity
Ear, Nose & Throat	D66B	Other Ear, Nose, Mouth and Throat Disorders, Minor Complexity
Orthopaedics	A11A	Insertion of Implantable Spinal Infusion Device, Major Complexity
Orthopaedics	A11B	Insertion of Implantable Spinal Infusion Device, Minor Complexity

Orthopaedics	B05Z	Carpal Tunnel Release
Orthopaedics	I01A	Bilateral and Multiple Major Joint Procedures of Lower Limb, Major Complexity
Orthopaedics	I01B	Bilateral and Multiple Major Joint Procedures of Lower Limb, Minor Complexity
Orthopaedics	I03A	Hip Replacement, Major Complexity
Orthopaedics	I03B	Hip Replacement, Minor Complexity
Orthopaedics	I04A	Knee Replacement, Major Complexity
Orthopaedics	I04B	Knee Replacement, Minor Complexity
Orthopaedics	I05A	Other Joint Replacement, Major Complexity
Orthopaedics	I05B	Other Joint Replacement, Minor Complexity
Orthopaedics	I06Z	Spinal Fusion for Deformity
Orthopaedics	I07Z	Amputation
Orthopaedics	I08A	Other Hip and Femur Procedures, Major Complexity
Orthopaedics	I08B	Other Hip and Femur Procedures, Minor Complexity
Orthopaedics	I11Z	Limb Lengthening Procedures
Orthopaedics	I12A	Misc Musculoskeletal Procs for Infect/Inflam of Bone/Joint, Major Complexity
Orthopaedics	I12B	Misc Musculoskeletal Procs for Infect/Inflam of Bone/Joint, Intermediate Comp
Orthopaedics	I12C	Misc Musculoskeletal Procs for Infect/Inflam of Bone/Joint, Minor Complexity
Orthopaedics	I13A	Humerus, Tibia, Fibula and Ankle Procedures, Major Complexity
Orthopaedics	I13B	Humerus, Tibia, Fibula and Ankle Procedures, Minor Complexity
Orthopaedics	I16Z	Other Shoulder Procedures
Orthopaedics	I18A	Other Knee Procedures, Major Complexity
Orthopaedics	I18B	Other Knee Procedures, Minor Complexity
Orthopaedics	I18Z	Other Knee Procedures
Orthopaedics	I19A	Other Elbow and Forearm Procedures, Major Complexity
Orthopaedics	I19B	Other Elbow and Forearm Procedures, Minor Complexity
Orthopaedics	I20A	Other Foot Procedures, Major Complexity
Orthopaedics	I20B	Other Foot Procedures, Minor Complexity
Orthopaedics	I20Z	Other Foot Procedures
Orthopaedics	I21Z	Local Excision and Removal of Internal Fixation Devices of Hip and Femur
Orthopaedics	I23A	Local Excision & Removal of Internal Fixation Device, Except Hip & Fmr, Maj Comp
Orthopaedics	I23B	Local Excision & Removal of Internal Fixation Device, Except Hip & Fmr, Min Comp
Orthopaedics	I23Z	Local Excision and Removal of Internal Fixation Devices, Except Hip and Femur
Orthopaedics	I24A	Arthroscopy, Major Complexity
Orthopaedics	I24B	Arthroscopy, Minor Complexity
Orthopaedics	I24Z	Arthroscopy
Orthopaedics	I25A	Bone and Joint Diagnostic Procedures Including Biopsy, Major Complexity
Orthopaedics	I25B	Bone and Joint Diagnostic Procedures Including Biopsy, Minor Complexity
Orthopaedics	I27A	Soft Tissue Procedures, Major Complexity
Orthopaedics	I27B	Soft Tissue Procedures, Minor Complexity
Orthopaedics	I28A	Other Musculoskeletal Procedures, Major Complexity
Orthopaedics	I28B	Other Musculoskeletal Procedures, Intermediate Complexity
Orthopaedics	I28C	Other Musculoskeletal Procedures, Minor Complexity
Orthopaedics	I29Z	Knee Reconstructions, and Revisions of Reconstructions

Orthopaedics	I30Z	Hand Procedures
Orthopaedics	I31A	Revision of Hip Replacement, Major Complexity
Orthopaedics	I31B	Revision of Hip Replacement, Intermediate Complexity
Orthopaedics	I31C	Revision of Hip Replacement, Minor Complexity
Orthopaedics	I32A	Revision of Knee Replacement, Major Complexity
Orthopaedics	I32B	Revision of Knee Replacement, Minor Complexity
Orthopaedics	I60Z	Femoral Shaft Fractures
Orthopaedics	I61A	Distal Femoral Fractures, Major Complexity
Orthopaedics	I61B	Distal Femoral Fractures, Minor Complexity
Orthopaedics	I63A	Sprains, Strains and Dislocations of Hip, Pelvis and Thigh, Major Complexity
Orthopaedics	I63B	Sprains, Strains and Dislocations of Hip, Pelvis and Thigh, Minor Complexity
Orthopaedics	I64A	Osteomyelitis, Major Complexity
Orthopaedics	I64B	Osteomyelitis, Minor Complexity
Orthopaedics	I67A	Septic Arthritis, Major Complexity
Orthopaedics	I67B	Septic Arthritis, Minor Complexity
Orthopaedics	I68A	Non-surgical Spinal Disorders, Major Complexity
Orthopaedics	I68B	Non-surgical Spinal Disorders, Minor Complexity
Orthopaedics	I69A	Bone Diseases and Arthropathies, Major Complexity
Orthopaedics	I69B	Bone Diseases and Arthropathies, Minor Complexity
Orthopaedics	I73A	Aftercare of Musculoskeletal Implants or Prostheses, Major Complexity
Orthopaedics	I73B	Aftercare of Musculoskeletal Implants or Prostheses, Minor Complexity
Orthopaedics	I74A	Injuries to Forearm, Wrist, Hand and Foot, Major Complexity
Orthopaedics	I74B	Injuries to Forearm, Wrist, Hand and Foot, Minor Complexity
Orthopaedics	I75A	Injuries to Shoulder, Arm, Elbow, Knee, Leg and Ankle, Major Complexity
Orthopaedics	I75B	Injuries to Shoulder, Arm, Elbow, Knee, Leg and Ankle, Minor Complexity
Orthopaedics	I77A	Fractures of Pelvis, Major Complexity
Orthopaedics	I77B	Fractures of Pelvis, Minor Complexity
Orthopaedics	I78A	Fractures of Neck of Femur, Major Complexity
Orthopaedics	I78B	Fractures of Neck of Femur, Minor Complexity
Orthopaedics	I79A	Pathological Fractures, Major Complexity
Orthopaedics	I79B	Pathological Fractures, Minor Complexity
Orthopaedics	I80Z	Femoral Fractures, Transferred to Acute Facility <2 Days
Orthopaedics	I81Z	Musculoskeletal Injuries, Sameday
Orthopaedics	I82Z	Other Sameday Treatment for Musculoskeletal Disorders
Orthopaedics	W02A	Hip, Femur and Lower Limb Procedures for Multiple Sig Trauma, Major Complexity
Orthopaedics	W02B	Hip, Femur and Lower Limb Procedures for Multiple Sig Trauma, Minor Complexity
Orthopaedics	X05A	Other Procedures for Injuries to Hand, Major Complexity
Orthopaedics	X05B	Other Procedures for Injuries to Hand, Minor Complexity
Ophthalmology	C01A	Procedures for Penetrating Eye Injury, Major Complexity
Ophthalmology	C01B	Procedures for Penetrating Eye Injury, Minor Complexity
Ophthalmology	C01Z	Procedures for Penetrating Eye Injury
Ophthalmology	C02Z	Enucleations and Orbital Procedures
Ophthalmology	C03A	Retinal Procedures, Major Complexity
Ophthalmology	C03B	Retinal Procedures, Minor Complexity
Ophthalmology	C03Z	Retinal Procedures
Ophthalmology	C04A	Major Corneal, Scleral and Conjunctival Procedures, Major Complexity
Ophthalmology	C04B	Major Corneal, Scleral and Conjunctival Procedures, Minor Complexity

Ophthalmology	C04Z	Major Corneal, Scleral and Conjunctival Procedures
Ophthalmology	C05Z	Dacryocystorhinostomy
Ophthalmology	C10Z	Strabismus Procedures
Ophthalmology	C11Z	Eyelid Procedures
Ophthalmology	C12Z	Other Corneal, Scleral and Conjunctival Procedures
Ophthalmology	C13Z	Lacrimal Procedures
Ophthalmology	C14A	Other Eye Procedures, Major Complexity
Ophthalmology	C14B	Other Eye Procedures, Minor Complexity
Ophthalmology	C14Z	Other Eye Procedures
Ophthalmology	C15Z	Glaucoma and Complex Cataract Procedures
Ophthalmology	C16Z	Lens Procedures
Ophthalmology	C60A	Acute and Major Eye Infections, Major Complexity
Ophthalmology	C60B	Acute and Major Eye Infections, Minor Complexity
Ophthalmology	C62A	Hyphaema and Medically Managed Trauma to the Eye, Major Complexity
Ophthalmology	C62B	Hyphaema and Medically Managed Trauma to the Eye, Minor Complexity
Ophthalmology	C63A	Other Disorders of the Eye, Major Complexity
Ophthalmology	C63B	Other Disorders of the Eye, Intermediate Complexity
Ophthalmology	C63C	Other Disorders of the Eye, Minor Complexity
Plastic & Reconstructive Surgery	B07A	Cranial or Peripheral Nerve and Other Nervous System Procedures, Major Comp
Plastic & Reconstructive Surgery	B07B	Cranial or Peripheral Nerve and Other Nervous System Procedures, Minor Comp
Plastic & Reconstructive Surgery	D03Z	Surgical Repair for Cleft Lip and Palate Disorders
Plastic & Reconstructive Surgery	D04A	Maxillo Surgery, Major Complexity
Plastic & Reconstructive Surgery	D04B	Maxillo Surgery, Minor Complexity
Plastic & Reconstructive Surgery	D04Z	Maxillo Surgery
Plastic & Reconstructive Surgery	D67A	Oral and Dental Disorders, Major Complexity
Plastic & Reconstructive Surgery	D67B	Oral and Dental Disorders, Minor Complexity
Plastic & Reconstructive Surgery	I02A	Microvascular Tissue Transfers or Skin Grafts, Excluding Hand, Major Complexity
Plastic & Reconstructive Surgery	I02B	Microvascular Tissue Transfers or Skin Grafts, Excluding Hand, Intermediate Comp
Plastic & Reconstructive Surgery	I02C	Microvascular Tissue Transfers or Skin Grafts, Excluding Hand, Minor Complexity
Plastic & Reconstructive Surgery	I17A	Maxillo-Facial Surgery, Major Complexity
Plastic & Reconstructive Surgery	I17B	Maxillo-Facial Surgery, Minor Complexity
Plastic & Reconstructive Surgery	I27C	Soft Tissue Procedures, Sameday
Plastic & Reconstructive Surgery	J01A	Microvas Tiss Transf for Skin, Subcut Tiss & Breast Dsrds, Major Complexity
Plastic & Reconstructive Surgery	J01B	Microvas Tiss Transf for Skin, Subcut Tiss & Breast Dsrds, Minor Complexity
Plastic & Reconstructive Surgery	J08A	Other Skin Grafts and Debridement Procedures, Major Complexity
Plastic & Reconstructive Surgery	J08B	Other Skin Grafts and Debridement Procedures, Intermediate Complexity

Plastic & Reconstructive Surgery	J08C	Other Skin Grafts and Debridement Procedures, Minor Complexity
Plastic & Reconstructive Surgery	J10A	Plastic OR Procs for Skin, Subcutaneous Tissue and Breast Disorders, Major Comp
Plastic & Reconstructive Surgery	J10B	Plastic OR Procs for Skin, Subcutaneous Tissue and Breast Disorders, Minor Comp
Plastic & Reconstructive Surgery	J10Z	Plastic OR Procedures for Skin, Subcutaneous Tissue and Breast Disorders
Plastic & Reconstructive Surgery	J11A	Other Skin, Subcutaneous Tissue and Breast Procedures, Major Complexity
Plastic & Reconstructive Surgery	J11B	Other Skin, Subcutaneous Tissue and Breast Procedures, Minor Complexity
Plastic & Reconstructive Surgery	J11Z	Other Skin, Subcutaneous Tissue and Breast Procedures
Plastic & Reconstructive Surgery	J12A	Lower Limb Procedures W Ulcer or Cellulitis, Major Complexity
Plastic & Reconstructive Surgery	J12B	Lower Limb Procedures W Ulcer or Cellulitis, Minor Complexity
Plastic & Reconstructive Surgery	J12C	Lower Limb Procs W Ulcer/Cellulitis W/O Cat CC W/O Skin Graft/Flap Repair
Plastic & Reconstructive Surgery	J13A	Lower Limb Procedures W/O Ulcer or Cellulitis, Major Complexity
Plastic & Reconstructive Surgery	J13B	Lower Limb Procedures W/O Ulcer or Cellulitis, Minor Complexity
Plastic & Reconstructive Surgery	J65C	Trauma to Skin, Subcutaneous Tissue and Breast, Sameday
Plastic & Reconstructive Surgery	K13Z	Plastic OR Procedures for Endocrine, Nutritional and Metabolic Disorders
Plastic & Reconstructive Surgery	X02A	Microvascular Tissue Transfer and Skin Grafts for Injuries to Hand, Major Comp
Plastic & Reconstructive Surgery	X02B	Microvascular Tissue Transfer and Skin Grafts for Injuries to Hand, Minor Comp
Plastic & Reconstructive Surgery	X07A	Skin Grafts for Injuries Excluding Hand, Major Complexity
Plastic & Reconstructive Surgery	X07B	Skin Grafts for Injuries Excluding Hand, Intermediate Complexity
Plastic & Reconstructive Surgery	X07C	Skin Grafts for Injuries Excluding Hand, Minor Complexity
Urology	K03Z	Adrenal Procedures
Urology	L04A	Kidney, Ureter and Major Bladder Procedures for Non-Neoplasm, Major Complexity
Urology	L04B	Kidney, Ureter and Major Bladder Procedures for Non-Neoplasm, Intermediate Comp
Urology	L04C	Kidney, Ureter and Major Bladder Procedures for Non-Neoplasm, Minor Complexity
Urology	L05A	Transurethral Prostatectomy for Urinary Disorder, Major Complexity
Urology	L05B	Transurethral Prostatectomy for Urinary Disorder, Minor Complexity
Urology	L06A	Minor Bladder Procedures, Major Complexity
Urology	L06B	Minor Bladder Procedures, Intermediate Complexity
Urology	L06C	Minor Bladder Procedures, Minor Complexity
Urology	L07A	Other Transurethral Procedures, Major Complexity
Urology	L07B	Other Transurethral Procedures, Minor Complexity
Urology	L08A	Urethral Procedures, Major Complexity
Urology	L08B	Urethral Procedures, Minor Complexity
Urology	L40Z	Ureteroscopy
Urology	L41Z	Cystourethroscopy for Urinary Disorder, Sameday
Urology	L42Z	ESW Lithotripsy

Urology	L64A	Urinary Stones and Obstruction, Major Complexity
Urology	L64B	Urinary Stones and Obstruction, Minor Complexity
Urology	L64C	Urinary Stones and Obstruction, Sameday
Urology	L65A	Kidney and Urinary Tract Signs and Symptoms, Major Complexity
Urology	L65B	Kidney and Urinary Tract Signs and Symptoms, Minor Complexity
Urology	L66Z	Urethral Stricture
Urology	M01A	Major Male Pelvic Procedures, Major Complexity
Urology	M01B	Major Male Pelvic Procedures, Minor Complexity
Urology	M02A	Transurethral Prostatectomy for Reproductive System Disorder, Major Complexity
Urology	M02B	Transurethral Prostatectomy for Reproductive System Disorder, Minor Complexity
Urology	M03A	Penis Procedures, Major Complexity
Urology	M03B	Penis Procedures, Minor Complexity
Urology	M03Z	Penis Procedures
Urology	M04Z	Testes Procedures
Urology	M06A	Other Male Reproductive System OR Procedures, Major Complexity
Urology	M06B	Other Male Reproductive System OR Procedures, Minor Complexity
Urology	M40Z	Cystourethroscopy for Male Reproductive System Disorder, Sameday
Urology	M61A	Benign Prostatic Hypertrophy, Major Complexity
Urology	M61B	Benign Prostatic Hypertrophy, Minor Complexity
Urology	M62A	Male Reproductive System Inflammation, Major Complexity
Urology	M62B	Male Reproductive System Inflammation, Minor Complexity
Urology	M63Z	Male Sterilisation Procedures
Urology	M64A	Other Male Reproductive System Disorders, Major Complexity
Urology	M64B	Other Male Reproductive System Disorders, Minor Complexity
Urology	M64Z	Other Male Reproductive System Disorders
Vascular Surgery	B04A	Extracranial Vascular Procedures, Major Complexity
Vascular Surgery	B04B	Extracranial Vascular Procedures, Intermediate Complexity
Vascular Surgery	B04C	Extracranial Vascular Procedures, Minor Complexity
Vascular Surgery	F08A	Major Reconstructive Vascular Procedures W/O CPB Pump, Major Complexity
Vascular Surgery	F08B	Major Reconstructive Vascular Procedures W/O CPB Pump, Intermediate Complexity
Vascular Surgery	F08C	Major Reconstructive Vascular Procedures W/O CPB Pump, Minor Complexity
Vascular Surgery	F11A	Amputation, Except Upper Limb and Toe, for Circulatory Disorders, Major Comp
Vascular Surgery	F11B	Amputation, Except Upper Limb and Toe, for Circulatory Disorders, Minor Comp
Vascular Surgery	F13A	Amputation, Upper Limb and Toe, for Circulatory Disorders, Major Complexity
Vascular Surgery	F13B	Amputation, Upper Limb and Toe, for Circulatory Disorders, Minor Complexity
Vascular Surgery	F14A	Vascular Procedures, Except Major Reconstruction, W/O CPB Pump, Major Complexity
Vascular Surgery	F14B	Vascular Procedures, Except Major Reconstruction, W/O CPB Pump, Interm Comp
Vascular Surgery	F14C	Vascular Procedures, Except Major Reconstruction, W/O CPB Pump, Minor Complexity
Vascular Surgery	F20Z	Vein Ligation and Stripping
Vascular Surgery	F21A	Other Circulatory System OR Procedures, Major Complexity
Vascular Surgery	F21B	Other Circulatory System OR Procedures, Intermediate Complexity
Vascular Surgery	F21C	Other Circulatory System OR Procedures, Minor Complexity

Vascular Surgery	F64A	Skin Ulcers in Circulatory Disorders, Major Complexity
Vascular Surgery	F64B	Skin Ulcers in Circulatory Disorders, Intermediate Complexity
Vascular Surgery	F64C	Skin Ulcers in Circulatory Disorders, Minor Complexity
Vascular Surgery	F65A	Peripheral Vascular Disorders, Major Complexity
Vascular Surgery	F65B	Peripheral Vascular Disorders, Minor Complexity
Vascular Surgery	J60A	Skin Ulcers, Major Complexity
Vascular Surgery	J60B	Skin Ulcers, Intermediate Complexity
Vascular Surgery	J60C	Skin Ulcers, Minor Complexity
Vascular Surgery	K01A	OR Procedures for Diabetic Complications, Major Complexity
Vascular Surgery	K01B	OR Procedures for Diabetic Complications, Intermediate Complexity
Vascular Surgery	K01C	OR Procedures for Diabetic Complications, Minor Complexity
Non Subspecialty Surgery	E66A	Major Chest Trauma, Major Complexity
Non Subspecialty Surgery	E66B	Major Chest Trauma, Minor Complexity
Non Subspecialty Surgery	E66C	Major Chest Trauma W/O CC
Non Subspecialty Surgery	G04A	Peritoneal Adhesiolysis, Major Complexity
Non Subspecialty Surgery	G04B	Peritoneal Adhesiolysis, Intermediate Complexity
Non Subspecialty Surgery	G04C	Peritoneal Adhesiolysis, Minor Complexity
Non Subspecialty Surgery	G07B	Appendicectomy, Minor Complexity
Non Subspecialty Surgery	G10A	Hernia Procedures, Major Complexity
Non Subspecialty Surgery	G10B	Hernia Procedures, Minor Complexity
Non Subspecialty Surgery	G12A	Other Digestive System OR Procedures, Major Complexity
Non Subspecialty Surgery	G12B	Other Digestive System OR Procedures, Intermediate Complexity
Non Subspecialty Surgery	G12C	Other Digestive System OR Procedures, Minor Complexity
Non Subspecialty Surgery	G65A	Gastrointestinal Obstruction, Major Complexity
Non Subspecialty Surgery	G65B	Gastrointestinal Obstruction, Minor Complexity
Non Subspecialty Surgery	G66A	Abdominal Pain and Mesenteric Adenitis, Major Complexity
Non Subspecialty Surgery	G66B	Abdominal Pain and Mesenteric Adenitis, Minor Complexity
Non Subspecialty Surgery	G70A	Other Digestive System Disorders, Major Complexity
Non Subspecialty Surgery	G70B	Other Digestive System Disorders, Minor Complexity
Non Subspecialty Surgery	G70C	Other Digestive System Disorders, Sameday
Non Subspecialty Surgery	I76A	Other Musculoskeletal Disorders, Major Complexity
Non Subspecialty Surgery	I76B	Other Musculoskeletal Disorders, Intermediate Complexity
Non Subspecialty Surgery	I76C	Other Musculoskeletal Disorders, Minor Complexity
Non Subspecialty Surgery	J65A	Trauma to Skin, Subcutaneous Tissue and Breast, Major Complexity
Non Subspecialty Surgery	J65B	Trauma to Skin, Subcutaneous Tissue and Breast, Minor Complexity
Non Subspecialty Surgery	K09A	Other Endocrine, Nutritional and Metabolic OR Procedures, Major Complexity
Non Subspecialty Surgery	K09B	Other Endocrine, Nutritional and Metabolic OR Procedures, Minor Complexity
Non Subspecialty Surgery	M05Z	Circumcision
Non Subspecialty Surgery	Q02A	Blood and Immune System Disorders W Other OR Procedures, Major Complexity
Non Subspecialty Surgery	Q02B	Blood and Immune System Disorders W Other OR Procedures, Minor Complexity
Non Subspecialty Surgery	T01A	Infectious and Parasitic Diseases W OR Procedures, Major Complexity
Non Subspecialty Surgery	T01B	Infectious and Parasitic Diseases W OR Procedures, Intermediate Complexity
Non Subspecialty Surgery	T01C	Infectious and Parasitic Diseases W OR Procedures, Minor Complexity
Non Subspecialty Surgery	T61A	Postoperative and Post-Traumatic Infections, Major Complexity
Non Subspecialty Surgery	T61B	Postoperative and Post-Traumatic Infections, Minor Complexity
Non Subspecialty Surgery	W04A	Multiple Significant Trauma W Other OR Procedures, Major Complexity
Non Subspecialty Surgery	W04B	Multiple Significant Trauma W Other OR Procedures, Minor Complexity

Non Subspecialty Surgery	W60Z	Multiple Trauma, Died or Transferred to Acute Facility <5 Days
Non Subspecialty Surgery	W61A	Multiple Significant Trauma W/O OR Procedures, Major Complexity
Non Subspecialty Surgery	W61B	Multiple Significant Trauma W/O OR Procedures, Minor Complexity
Non Subspecialty Surgery	X04A	Other Procedures for Injuries to Lower Limb, Major Complexity
Non Subspecialty Surgery	X04B	Other Procedures for Injuries to Lower Limb, Minor Complexity
Non Subspecialty Surgery	X06A	Other Procedures for Other Injuries, Major Complexity
Non Subspecialty Surgery	X06B	Other Procedures for Other Injuries, Intermediate Complexity
Non Subspecialty Surgery	X06C	Other Procedures for Other Injuries, Minor Complexity
Non Subspecialty Surgery	X40Z	Injuries, Poisoning and Toxic Effects of Drugs W Ventilator Support
Non Subspecialty Surgery	X60A	Injuries, Major Complexity
Non Subspecialty Surgery	X60B	Injuries, Minor Complexity
Non Subspecialty Surgery	X63A	Sequelae of Treatment, Major Complexity
Non Subspecialty Surgery	X63B	Sequelae of Treatment, Minor Complexity
Non Subspecialty Surgery	Y03Z	Other OR Procedures for Other Burns
Non Subspecialty Surgery	Y62A	Other Burns, Major Complexity
Non Subspecialty Surgery	Y62B	Other Burns, Minor Complexity
Non Subspecialty Surgery	Y62C	Other Burns, Sameday
Non Subspecialty Surgery	Z01A	Other Contacts W Health Services W OR Procedures, Major Complexity
Non Subspecialty Surgery	Z01B	Other Contacts W Health Services W OR Procedures, Minor Complexity
Transplantation	A01Z	Liver Transplant
Transplantation	A03Z	Lung or Heart-Lung Transplant
Transplantation	A05Z	Heart Transplant
Transplantation	A07A	Allogeneic Bone Marrow Transplant, Age <=16 Years or Major Complexity
Transplantation	A07B	Allogeneic Bone Marrow Transplant, Age >=17 Years and Minor Complexity
Transplantation	A08A	Autologous Bone Marrow Transplant, Major Complexity
Transplantation	A08B	Autologous Bone Marrow Transplant, Minor Complexity
Transplantation	A09A	Kidney Transplant, Age <=16 Years or Major Complexity
Transplantation	A09B	Kidney Transplant, Age >=17 Years and Minor Complexity
Extensive Burns	Y01Z	Vent >=96hrs or Trach for Burns or OR Procs for Severe Full Thickness Burns
Extensive Burns	Y02A	Skin Grafts for Other Burns, Major Complexity
Extensive Burns	Y02B	Skin Grafts for Other Burns, Intermediate Complexity
Extensive Burns	Y02C	Skin Grafts for Other Burns, Minor Complexity
Extensive Burns	Y03A	Other OR Procedures for Other Burns, Major Complexity
Extensive Burns	Y03B	Other OR Procedures for Other Burns, Minor Complexity
Extensive Burns	Y60Z	Burns, Transferred to Acute Facility <5 Days
Extensive Burns	Y61Z	Severe Burns
Tracheostomy	A06A	Tracheostomy and/or Ventilation >=96hours, Major Complexity
Tracheostomy	A06B	Tracheostomy and/or Ventilation >=96hours, Intermediate Complexity
Tracheostomy	A06C	Tracheostomy and/or Ventilation >=96hours, Minor Complexity
Gynaecology	N01A	Pelvic Evisceration and Radical Vulvectomy, Major Complexity
Gynaecology	N01B	Pelvic Evisceration and Radical Vulvectomy, Minor Complexity
Gynaecology	N04A	Hysterectomy for Non-Malignancy, Major Complexity
Gynaecology	N04B	Hysterectomy for Non-Malignancy, Minor Complexity
Gynaecology	N05A	Oophorectomy and Complex Fallopian Tube Procedures for Non-Malignancy, Maj Comp
Gynaecology	N05B	Oophorectomy and Complex Fallopian Tube Procedures for Non-Malignancy, Min Comp
Gynaecology	N06A	Female Reproductive System Reconstructive Procedures, Major Complexity

Gynaecology	N06B	Female Reproductive System Reconstructive Procedures, Minor Complexity
Gynaecology	N06Z	Female Reproductive System Reconstructive Procedures
Gynaecology	N07A	Other Uterus and Adnexa Procedures for Non-Malignancy, Major Complexity
Gynaecology	N07B	Other Uterus and Adnexa Procedures for Non-Malignancy, Minor Complexity
Gynaecology	N08Z	Endoscopic and Laparoscopic Procedures, Female Reproductive System
Gynaecology	N09Z	Other Vagina, Cervix and Vulva Procedures
Gynaecology	N10Z	Diagnostic Curettage and Diagnostic Hysteroscopy
Gynaecology	N11A	Other Female Reproductive System OR Procedures, Major Complexity
Gynaecology	N11B	Other Female Reproductive System OR Procedures, Minor Complexity
Gynaecology	N11Z	Other Female Reproductive System OR Procedures
Gynaecology	N61A	Female Reproductive System Infections, Major Complexity
Gynaecology	N61B	Female Reproductive System Infections, Minor Complexity
Gynaecology	N61Z	Female Reproductive System Infections
Gynaecology	N62A	Menstrual and Other Female Reproductive System Disorders, Major Complexity
Gynaecology	N62B	Menstrual and Other Female Reproductive System Disorders, Minor Complexity
Gynaecology	N62Z	Menstrual and Other Female Reproductive System Disorders
Gynaecology	O03A	Ectopic Pregnancy, Major Complexity
Gynaecology	O03B	Ectopic Pregnancy, Minor Complexity
Gynaecology	O04A	Postpartum and Post Abortion W OR Procedures, Major Complexity
Gynaecology	O04B	Postpartum and Post Abortion W OR Procedures, Minor Complexity
Gynaecology	O04C	Postpartum and Post Abortion W OR Procedures, Sameday
Gynaecology	O05Z	Abortion W OR Procedures
Gynaecology	O63A	Abortion W/O OR Procedures, Major Complexity
Gynaecology	O63B	Abortion W/O OR Procedures, Minor Complexity
Obstetrics	O01A	Caesarean Delivery, Major Complexity
Obstetrics	O01B	Caesarean Delivery, Intermediate Complexity
Obstetrics	O01C	Caesarean Delivery, Minor Complexity
Obstetrics	O02A	Vaginal Delivery W OR Procedures, Major Complexity
Obstetrics	O02B	Vaginal Delivery W OR Procedures, Minor Complexity
Obstetrics	O60A	Vaginal Delivery, Major Complexity
Obstetrics	O60B	Vaginal Delivery, Intermediate Complexity
Obstetrics	O60C	Vaginal Delivery, Minor Complexity
Obstetrics	O61A	Postpartum and Post Abortion W/O OR Procedures, Major Complexity
Obstetrics	O61B	Postpartum and Post Abortion W/O OR Procedures, Minor Complexity
Obstetrics	O61Z	Postpartum and Post Abortion W/O OR Procedures
Obstetrics	O63Z	Abortion W/O OR Procedures
Obstetrics	O66A	Antenatal and Other Obstetric Admissions, Major Complexity
Obstetrics	O66B	Antenatal and Other Obstetric Admissions, Minor Complexity
Obstetrics	O66C	Antenatal and Other Obstetric Admissions, Sameday
Drug & Alcohol	V60A	Alcohol Intoxication and Withdrawal, Major Complexity
Drug & Alcohol	V60B	Alcohol Intoxication and Withdrawal, Minor Complexity
Drug & Alcohol	V61A	Drug Intoxication and Withdrawal, Major Complexity
Drug & Alcohol	V61B	Drug Intoxication and Withdrawal, Minor Complexity
Drug & Alcohol	V61Z	Drug Intoxication and Withdrawal
Drug & Alcohol	V62A	Alcohol Use and Dependence, Major Complexity
Drug & Alcohol	V62B	Alcohol Use and Dependence, Minor Complexity
Drug & Alcohol	V62Z	Alcohol Use and Dependence

Drug & Alcohol	V63Z	Opioid Use and Dependence
Drug & Alcohol	V64Z	Other Drug Use and Dependence
Drug & Alcohol	V65Z	Treatment for Alcohol Disorders, Sameday
Drug & Alcohol	V66Z	Treatment for Drug Disorders, Sameday
Drug & Alcohol	X40A	Injuries, Poisoning and Toxic Effects of Drugs W Ventilator Support, Major Comp
Drug & Alcohol	X40B	Injuries, Poisoning and Toxic Effects of Drugs W Ventilator Support, Minor Comp
Drug & Alcohol	X62A	Poisoning/Toxic Effects of Drugs and Other Substances, Major Complexity
Drug & Alcohol	X62B	Poisoning/Toxic Effects of Drugs and Other Substances, Minor Complexity
Psychiatry	U40Z	Mental Health Treatment W ECT, Sameday
Psychiatry	U60A	Mental Health Treatment W/O ECT, Sameday, Major Complexity
Psychiatry	U60B	Mental Health Treatment W/O ECT, Sameday, Minor Complexity
Psychiatry	U60Z	Mental Health Treatment W/O ECT, Sameday
Psychiatry	U61A	Schizophrenia Disorders, Major Complexity
Psychiatry	U61B	Schizophrenia Disorders, Minor Complexity
Psychiatry	U62A	Paranoia and Acute Psychotic Disorders, Major Complexity
Psychiatry	U62B	Paranoia and Acute Psychotic Disorders, Minor Complexity
Psychiatry	U63A	Major Affective Disorders, Major Complexity
Psychiatry	U63B	Major Affective Disorders, Minor Complexity
Psychiatry	U64A	Other Affective and Somatoform Disorders, Major Complexity
Psychiatry	U64B	Other Affective and Somatoform Disorders, Minor Complexity
Psychiatry	U64Z	Other Affective and Somatoform Disorders
Psychiatry	U65A	Anxiety Disorders, Major Complexity
Psychiatry	U65B	Anxiety Disorders, Minor Complexity
Psychiatry	U65Z	Anxiety Disorders
Psychiatry	U66A	Eating and Obsessive-Compulsive Disorders, Major Complexity
Psychiatry	U66B	Eating and Obsessive-Compulsive Disorders, Minor Complexity
Psychiatry	U66Z	Eating and Obsessive-Compulsive Disorders
Psychiatry	U67A	Personality Disorders and Acute Reactions, Major Complexity
Psychiatry	U67B	Personality Disorders and Acute Reactions, Minor Complexity
Psychiatry	U67Z	Personality Disorders and Acute Reactions
Unallocated	801A	OR Procedures Unrelated to Principal Diagnosis, Major Complexity
Unallocated	801B	OR Procedures Unrelated to Principal Diagnosis, Intermediate Complexity
Unallocated	801C	OR Procedures Unrelated to Principal Diagnosis, Minor Complexity
Unallocated	960Z	Ungroupable
Unallocated	961Z	Unacceptable Principal Diagnosis

Table S2: Uni-variate associations with prolonged hospital length of stay for patients hospitalised over two years in Victoria, Australia

Variable	Overall	Length of stay (days)		P-value*
		1-13	≥14	
Number of cases	1696112	1532662 (90.4)	163450 (9.6)	
Female (n, %)	877020 (51.7)	788874 (51.5)	88146 (53.9)	<0.001
Number (%) aged ≥65 years	881090 (51.9)	770683 (50.3)	110227 (67.4)	<0.001
Married or defacto relationship	955280 (56.3)	882026 (57.6)	73254 (44.8)	<0.001
Indigenous population	13888 (0.8)	12733 (0.8)	1155 (0.7)	<0.001
Charlson co-morbidity Index* n (%)				
0	978956 (57.7)	916511 (59.8)	62445 (38.2)	
1-2	485591 (28.6)	426423 (27.8)	59168 (36.2)	
3-4	114398 (6.7)	92390 (6.0)	22008 (13.5)	
5-6	24080 (1.4)	19012 (1.2)	5068 (3.1)	
>6	93087 (5.5)	78326 (5.1)	14761 (9.0)	<0.001
Current smoker n(%)	182178 (10.7)	164263 (10.7)	17915 (11.0)	0.003
DRG (Medical)	1044369 (61.6)	916416 (59.8)	127953 (78.3)	<0.001
Emergency (unscheduled) admission n (%)	839360 (49.5)	783477 (51.1)	55883 (34.2)	<0.001
ICU Stay	79,115 (4.7)	61027 (4.0)	18088 (11.1)	<0.001
No. of admissions past 12 months n (%)				
0	711166 (41.9)	676363 (44.1)	34803 (21.3)	
1	374576 (22.1)	334055 (21.8)	40521 (24.8)	
≥2	610370 (36.0)	522244 (34.1)	88126 (53.9)	<0.001
Admission source, n (%)				
Home	1442065 (85.0)	1355996 (88.5)	86069 (52.7)	
Age care residential facility	11302 (0.7)	10317 (0.7)	985 (0.6)	
From another hospital	195230 (11.5)	136540 (8.9)	58690 (35.9)	
Other	47515 (2.8)	29809 (1.9)	17706 (10.8)	<0.001
Admitted on a weekend, n (%)	573292 (33.8)	515274 (33.6)	58018 (35.5)	<0.001
Season n (%)				
Spring (Sep-Nov)	406209 (24.0)	370344 (24.1)	35865 (21.9)	
Summer (Dec-Feb)	415172 (24.5)	374786 (24.5)	40386 (24.7)	
Autumn (Mar-May)	433328 (25.6)	390402 (25.50	42926 (26.3)	
Winter (Jun-Aug)	441403 (26.0)	397130 (25.9)	44273 (27.1)	<0.001
Risk category based on admission unit n (%)				
1	659004 (38.9)	633300 (41.3)	25704 (15.7)	
2	503703 (29.7)	451536 (29.5)	52167 (31.9)	
3	387474 (22.8)	344182 (22.5)	43292 (26.5)	
4	145931 (8.6)	103644 (6.7)	42287 (25.9)	<0.001

DRG = diagnostic related group, ICU = intensive care unit; *based on chi-squared tests

Table S3: Associations with prolonged hospitalization (≥14 days) for patients hospitalized over two years in Victoria, Australia who were discharged alive

Variable	Longer LOS (odds ratio [OR], 95% CI)
Age groups, years.	
20-24	0.74 (0.71-0.77)
25-29	0.84 (0.81-0.88)
30-34	0.97 (0.94-1.02)
35-39	1.01 (0.97-1.06)
40-44	0.98 (0.94-1.02)
45-49	0.96 (0.93-0.99)
50-54	0.96 (0.93-0.99)
55-59	0.95 (0.92-0.98)
60-64	reference
65-69	1.02 (0.99-1.05)
70-74	1.16 (1.12-1.19)
75-79	1.48 (1.44-1.52)
80-84	1.82 (1.76-1.87)
≥85	2.38 (2.32-2.45)
Female gender	1.09 (1.08-1.11)
Indigenous population	
Yes	0.90 (0.84-0.96)
No	1.0 [reference]
Undefined	1.15 (1.09-1.21)
Charlson co-morbidity Index* n	
0	1.0 [reference]
1-2	1.44 (1.42-1.46)
3-4	2.06 (2.02-2.10)
5-6	2.20 (2.12-2.29)
>6	0.92 (0.90-0.95)
Marital Status	
Never married	1.60 (1.57-1.63)
Widowed	1.27 (1.25-1.29)
Married or defacto	1.0 [reference]
Divorced or separated	1.38 (1.35-1.41)
Other	1.32 (1.27-1.38)
Current smoker	1.27 (1.24-1.29)
DRG type	
Medical	1.0 [reference]
Surgical	0.60 (0.59-0.61)
Other	0.79 (0.76-0.82)
Emergency (unscheduled) admission	0.64 (0.63-0.65)
Admission source	
Home	1.0 [reference]
Aged care residential facility	1.03 (0.96-1.10)
From another hospital	4.35 (4.28-4.42)
Other	4.85 (4.73-4.97)
ICU stay	3.46 (3.38-3.54)
Admitted on weekend	1.07 (1.05-1.08)
Season of admission	
Spring (Sep-Nov)	1.0 [reference]
Summer (Dec-Feb)	1.17 (1.15-1.19)
Autumn (Mar-May)	1.16 (1.14-1.18)
Winter (Jun-Aug)	1.15 (1.13-1.17)
Prior admissions (in 12 months)	
0	reference
1	1.20 (1.18-1.22)
≥2	1.30 (1.28-1.32)

Risk category based on admission unit	
1	1.0 [reference]
2	1.88 (1.85-1.91)
3	2.45 (2.41-2.49)
4	8.97 (8.79-9.16)

Table S4: Uni-variate associations with in-hospital mortality for patients hospitalized over two years in Victoria, Australia

Variable	Overall	Death		P-value*
		No	Yes	
Number of cases	1696112 (100.0)	1660825 (97.9)	35287 (2.1)	-
Female (n, %)	877020 (51.7)	860667 (51.8)	16353 (46.3)	<0.001
Number (%) aged ≥65 years	881090 (51.9)	851853 (51.3)	29237 (82.9)	<0.001
Married or defacto relationship	955280 (56.3)	936807 (56.4)	18473 (52.4)	<0.001
Indigenous population	13888 (0.8)	13706 (0.8)	182 (0.5)	<0.001
Charlson co-morbidity Index* n (%)				
0	978956 (57.7)	975446 (58.7)	3510 (10.0)	
1-2	485591 (28.6)	474109 (28.6)	11482 (32.5)	
3-4	114398 (6.7)	108023 (6.5)	6375 (18.1)	
5-6	24080 (1.4)	21967 (1.3)	2113 (6.0)	
>6	93087 (5.5)	81280 (4.9)	11807 (33.5)	<0.001
Current smoker n (%)	182178 (10.7)	180043 (10.8)	235 (6.1)	<0.001
DRG (Medical)	1044369 (61.6)	1014769 (61.1)	29600 (83.9)	<0.001
Emergency (unscheduled) admission n (%)	839360 (49.5)	820075 (49.4)	19285 (54.7)	<0.001
ICU Stay	79115 (4.7)	73424 (4.4)	5691 (16.1)	<0.001
No. of admissions past 12 months n (%)				
0	711166 (41.9)	704000 (42.4)	7166 (20.3)	
1	374576 (22.1)	368553 (22.2)	6023 (17.1)	
≥2	610370 (36.0)	588272 (35.4)	22098 (62.6)	<0.001
Admission source, n (%)				
Home	1442065 (85.0)	1419584 (85.5)	22481 (63.7)	
Age care residential facility	11302 (0.7)	10418 (0.6)	884 (2.5)	
From another hospital	195230 (11.5)	187394 (11.3)	7836 (22.2)	
Other	47515 (2.8)	43429 (2.6)	4086 (11.6)	<0.001
Admitted on a weekend, n (%)	573292 (33.8)	562001 (33.8)	11291 (32.0)	<0.001
Season n (%)				
Spring (Sep-Nov)	406209 (24.0)	398017 (24.0)	8192 (23.2)	
Summer (Dec-Feb)	415172 (24.5)	406962 (24.5)	8210 (23.3)	
Autumn (Mar-May)	433328 (25.5)	424127 (25.5)	9201 (26.1)	
Winter (Jun-Aug)	441403 (26.0)	431719 (26.0)	9684 (27.4)	<0.001
Risk category based on admission unit, n (%)				
1	659004 (38.9)	653279 (39.3)	5725 (16.2)	
2	503703 (29.7)	489964 (29.5)	13739 (38.9)	
3	387474 (22.8)	383104 (23.1)	4370 (12.4)	
4	145931 (8.6)	134478 (8.1)	11453 (32.5)	<0.001
Median LOS (IQR)	3 (1-6)	3 (1-6)	6 (2-13)	<0.001

DRG = diagnostic related group, ICU = intensive care unit, LOS = length of stay; *based on chi-squared tests

Table S5 Nature and destination of discharge among people who survived in hospital for patients hospitalized over two years in Victoria, Australia

Discharge destination	Number (%)
All alive	1660825 (100)
Discharge and transfer to mental health residential facility	2814 (0.2)
Discharge and transfer to Transition Care bed-based program	7775 (0.5)
Posthumous Organ Procurement	107 (0.01)
Discharge to private residence/accommodation	1388194 (83.6)
Discharge and transfer to aged care residential facility	42051 (2.5)
Statistical discharge (change in Care Type within this hospital)	47877 (2.9)
Discharge and transfer to other acute hospital/extended care/rehabilitation/geriatric centre	159160 (9.6)
Left against medical advice	12847 (0.8)

Table S6: Independent associations with hospital discharge to home for patients hospitalised over two years in Victoria, Australia

Variable	(odds ratio [OR], 95% CI)
Age groups, yrs.	
20-24	2.16 (2.08-2.25)
25-29	2.01 (1.94-2.08)
30-34	1.83 (1.77-1.90)
35-39	1.60 (1.55-.166)
40-44	1.58 (1.53-1.63)
45-49	1.51 (1.46-1.56)
50-54	1.35 (1.32-1.39)
55-59	1.19 (1.16-1.22)
60-64	reference
65-69	0.83 (0.81-0.85)
70-74	0.68 (0.67-0.70)
75-79	0.54 (0.53-0.55)
80-84	0.42 (0.41-0.43)
≥85	0.30 (0.29-0.31)
Female gender	0.94 (0.93-0.95)
Indigenous population	
Yes	0.75 (0.71-0.79)
No	1.0 [reference]
Undefined	0.98 (0.93-1.02)
Charlson co-morbidity Index* n	
0	1.0 [reference]
1-2	0.75 (0.74-0.76)
3-4	0.57 (0.56-0.58)
5-6	0.50 (0.48-0.52)
>6	0.59 (0.58-0.60)
Marital Status	
Never married	0.61 (0.60-0.61)
Widowed	0.75 (0.74-0.76)
Married or defacto	1.0 [reference]
Divorced or separated	0.67 (0.66-0.68)
Other	0.69 (0.67-0.72)
Current smoker	0.88 (0.86-0.89)
DRG type	
Medical	1.0 [reference]
Surgical	0.95 (0.94-0.96)
Other	1.25 (1.22-1.29)
Emergency (unscheduled) admission	0.42 (0.41-0.43)
Admission source	
Home	1.0 [reference]
Aged care residential facility	0.13 (0.12-0.13)
From another hospital	0.48 (0.47-0.48)
Other	0.33 (0.32-0.34)
ICU stay	0.40 (0.39-0.42)
Admitted on weekend	1.00 (0.99-1.01)
Season of admission	
Spring (Sep-Nov)	1.0 [reference]
Summer (Dec-Feb)	0.96 (0.94-0.97)
Autumn (Mar-May)	0.96 (0.94-0.97)
Winter (Jun-Aug)	0.98 (0.96-0.99)
Prior admissions (in 12 months)	
0	reference
1	1.13 (1.12-1.15)
≥2	0.94 (0.92-0.95)

Risk category based on admission unit	
1	1.0 [reference]
2	0.62 (0.61-0.63)
3	0.34 (0.34-0.35)
4	0.49 (0.38-0.50)
Longer length of stay (≥14 days)	0.53 (0.52-0.54)

DRG = diagnostic related group, ICU = intensive care unit, LOS = length of stay

Table S7: Summary of literature describing epidemiology of prolonged hospitalisations 2015 to 2019.

Details of study	Cohort and setting	Details of long stay patients LOS = length of stay	Baseline variables and complications associated with long stay
Marfil-Garza et al<br><br>2018<br><br>Mexico	85904 hospitalisations between Jan 2000 and Dec 2017 with LOS $\geq$ one day<br>Single centre study<br>Public tertiary in Mexico city<br>167 hospital beds with 4772 admissions annually<br>Median age = 51 (35-66) yr<br>Surgery performed 41.8%<br>55.6% female	Prolonged LOS was > 95th centile ($\geq$ 34 days)<br><br>Overall median hospital LOS = 8 (5-14) days<br><br>5.1% of admissions had prolonged LOS<br>Prolonged LOS used 23.1% of total bed days<br><br>The risk of mortality of prolonged LOS was 13.3% vs 3.7%	Prolonged LOS was associated with<br>• Younger age<br>• Male gender<br>• Emergency and weekend admission<br>• Surgery<br>• Number of co-morbidities<br>• Lower socio-economic status<br>• Lower patient-to-physician ratio<br>• Bone marrow transplantation<br>• Complex infectious conditions<br>• Intestinal fistulae
Lisk et al<br><br>2019<br><br>United Kingdom<br>Surrey	374 patients with 23 acute conditions<br><br>Single centre study<br><br>Median age 85 (78-90) yr<br>34% men	Prolonged LOS = LOS > 5th quintile (17 days)<br><br>Median LOS = 6.5 (1.8-14.8) days<br>Mortality = 10.2% overall<br><br>Prolonged LOS was associated with discharge to places other than usual residence = OR 3.1 (1.7-5.7)	Prolonged LOS was associated with<br><br>• Acute stroke<br>• Acute first hip fracture<br>• Recurrent hip fracture<br>• Urinary tract infection/pneumonia<br>• Acute malignancy
Crisafulli et al<br><br>2018<br><br>Spain	May 2009 to May 2016<br>437 patients with exacerbations of COAD<br>Single centre study<br>Median age approx 72 yr<br>Approx. 80% male	LOS longer than 7 days<br>224 (51.2%) had LOS > 7 days<br><br>Increase ventilation and ICU admission<br>Increased 6 month, 1 year and 3 year mortality	Prolonged LOS was associated with<br>• mMRC dyspnoea score $\geq$ 2<br>• acute respiratory acidosis
Thomas et al<br><br>2018<br><br>Philadelphia USA	Retrospective study 2012 to 2014<br><br>Multi-centre study<br>1115 cases of head and neck malignancy with free tissue transfer flap<br><br>33.2% female<br>Mean (SD) age 66.8 (3.9) yr	Prolonged LOS at least 13 days (top quartile of duration of LOS)	Prolonged LOS was associated with<br>• Return to the operating room<br>• Smoking<br>• Clean-contaminated wound<br>• Age<br>• Bony flap<br>• Operative time
Nguyen et al<br><br>2019<br><br>Vietnam	461 elderly patients admitted to hospital<br>Single centre study<br>Mean age 76.2 $\pm$ 8.9 yr<br>56.8% female	Prolonged hospitalisation = exceeded 75th centile (> 18 days)<br><br>Prolonged hospitalisation was seen in 106 / 461 = 23.0%	Prolonged LOS was associated with<br>• Clinical frailty
Agosti et al<br><br>2018<br><br>Italy and Spain	1894 patients 2012-2016<br>Acutely admitted elderly patients<br>Multi-centre study<br><br>Mean age approx. 79 yr<br>48.6% male	Study examined associations with living alone after adjusting for other variables<br><br>Mean length of stay =<br>• 13.1 $\pm$ 11.6 days (living alone)<br>11.6 $\pm$ 9.3 days (not living alone)	In four different statistical modules living alone was associated with longer hospital LOS
Rana et al<br><br>Houston, USA<br><br>2017	73635 liver transplants March 2002 to June 2016<br>In organ procurement and transplantation network<br><br>Multi-centre study<br><br>Excluded multi-visceral transplant and deaths within one month<br><br>33.0% female	Prolonged hospital stay > 30 days<br><br>Mean hospital stay = 16.4 days	Prolonged LOS was associated with<br>• ICU admission<br>• Previous transplant<br>• Hospital admission<br>• Ventilator<br>• Dialysis<br>• Life support<br>• Encephalopathy<br>• TIPS<br>• Ascites<br>• Previous surgery

	Mean (SD) age = 54.3 ± 10.3 yr		• Increasing age<br>• Increasing MELD<br>• Low serum Albumin<br>High serum Na
Coelho et al<br><br>2018<br><br>Brasil	1011 patients undergoing retropubic radical prostatectomy Jan 2010 to Jan 2012<br><br>Single centre study<br><br>Median (IQR) age = 65.4 (60.2-69.7) years	Median LOS = 2 days<br>217 (21.5%) of patients had prolonged hospitalisation (> 2days)	Prolonged LOS was associated with<br>• CCI<br>• Age<br>• ASA score of 3<br>• Prostate volume<br>• Operative time<br><br>• Presence of any complications<br>• Major complications
Wasfy et al<br><br>2018<br><br>Boston, USA	906324 patients discharged alive for acute myocardial infarction between Jul 2008 and Mar 2017<br>Multi-centre study<br>Mean age approx. 64 yr<br>Approx. 2/3 male	16.8% had prolonged LOS (≥ 7 days)	Prolonged LOS was associated with<br>• Older age<br>• Heart failure on admission<br>• Higher heart rate on admission<br>• Systolic blood pressure on admission<br>• Shock on admission<br>• Lower glomerular filtration rate<br>Lower haemoglobin
O'Sullivan et al<br><br>2017<br><br>Melbourne, Australia	15623 adult (≥ 18 years) patients and 22094 multi-day admissions Jul 2013 to June 2014<br><br>Single centre study<br><br>Median age = 65 (47-78) yr<br>47.4% female	Median LOS 4 (2-8) days<br><br>Long stay (≥14 days) comprised 13.1 % multi-day admissions but used 49.1% of bed days	Prolonged LOS was associated with<br>• Multiple admission in the study period<br>• Admission from another hospital<br>• CCI<br>• History of smoking<br>• Nature of the admitted unit<br>• ICU admission<br>• Longer duration mechanical ventilation<br>• More frequent Medical emergency team review<br>Increased risk of in-hospital death (8.2 vs 3.1%) and less likely to be discharged home.
Tsilimigras et al<br><br>2019<br><br>15 hospitals in US, Italy, Romania, Canada, France, Portugal, Japan, Australia, China, Netherlands.	706 patients undergoing hepatectomy for intra-hepatic cholangiocarcinoma between 1990 and 2016<br>Multi-centre study<br><br>Median age = 57.9 (49.3-66.0) years<br>59.4% male	Prolonged LOS defined as > 10 days	Prolonged LOS was associated with<br>Increased Albumin-BR grade
Almogati et al<br><br>2019<br><br>Saudi Arabia	305 patients admitted for coronary artery bypass surgery Nov 2013 to May 2015<br><br>Single centre study<br><br>Mean age = 59.1years<br>Range = 31-87 years<br><br>82% male	LOS ≥ 14 days proposed as an extended LOS<br>= 64/305 (21.0%)<br><br>mean LOS = 11.8 ± 11.9 days<br><br>81.6% of patients had diabetes mellitus<br><br>64% had HbA1C ≥ 7%	Prolonged LOS was associated with left ventricular ejection fraction<br><br>Prolonged LOS was not associated with<br>• Increasing age<br>• Gender<br>• Hypertension<br>• Dyslipidemia
Agrawal et al<br><br>2018<br><br>USA	157589 women who underwent hysterectomy<br>52.8% minimally invasive<br>28.6% abdominal<br>18.6% vaginal<br><br>Multi-centre study<br><br>Approx. 90% less than 59 yr	LOS above 75th centile<br>• 1 day for minimally invasive<br>• 3 days for abdominal<br>2 days for vaginal	Baseline factors associated with prolonged LOS.<br>• Increasing age<br>• Diabetes<br>• Chronic obstructive airways disease<br>• Bleeding disorders<br>• Body mass index<br>• Functional status<br>• Total operative time

			Development of post-operative complications (wound infection, pneumonia, pulmonary embolism, deep venous thrombosis, urinary tract infection, sepsis, reoperation)
Fletcher et al<br><br>2018<br><br>USA	11430 patients undergoing laparoscopic sleeve gastrectomy between 2009 and 2012<br><br>Multi-centre study<br><br>Approx. ¾ female<br><br>More than 96% aged < 65 yr	Prolonged LOS defined as $\geq$ 3 days which occurred in 18.4%<br><br>Median LOS = 2 days	Independent associations with prolonged LOS included:<br>• Age > 65 yr<br>• Female gender<br>• Body mass index > 50<br>• Chronic obstructive pulmonary disease<br>• Hypertension<br>• Renal insufficiency<br>• Anaemia<br>Prolonged operative time
Agrawal et al<br><br>2018<br><br>USA	19084 women who underwent hysterectomy for uterine cancer from 2006 to 2015<br><br>31.9% abdominal<br>68.1% minimally invasive<br><br>Multi-centre study<br><br>Approx. 60% aged over 60 yrs	LOS above 75th centile<br>• 1 day for minimally invasive<br>• 5 days for abdominal<br><br>The aim was to identify what proportion of prolonged LOS could be accounted for by the measured variables	In the abdominal group risk factors combined accounted for 23.6% of the variation in prolonged LOS. Demographic characteristics explained 4.0%, preoperative factors 7.0%, intraoperative factors 7.9%, and postoperative characteristics 9.7%.<br><br>In the minimally invasive group, the 75th and 90th percentiles for LOS were 1 and 2 days, respectively. The combined risk factors explained 16.2% of the variation in prolonged LOS. Demographic characteristics accounted for 6.2%, preoperative factors 4.1%, intraoperative factors 6.9%, and postoperative characteristics 1.3%.
Lee et al<br><br>2018<br><br>Singapore	Adult surgical patients with hepato-biliary malignancy (150) and neurosurgery (166) between Jan 2014 and Jan 2015<br><br>Single centre study<br><br>Male gender approx. 40%<br>Mean age approx. 60 yrs	Prolonged LOS defined as above median LOS<br><br>Median LOS<br>• 6 (2-8) days for hepato-biliary cancer<br>6 (4-9) days for neurosurgery	Variables independently associated with prolonged LOS for hepato-biliary malignancy<br>• Increasing age<br>• Admission after 5pm<br>• Operation duration<br>Additional variables associated with prolonged LOS for neurosurgery<br>• Functional status<br>• Number of hospital-acquired infections<br>• Referral to occupational therapy
Crawford et al<br><br>2018<br><br>USA	16723 patients undergoing heart transplantation between 2003 and 2012<br><br>Multi-centre study<br><br>Median age 55 (45-61) years<br><br>76% male	Prolonged LOS defined as > 30 days<br><br>Mean (SD) LOS = 19 $\pm$ 21 days<br><br>12.1% had LOS > 30 days	Variables independently associated with prolonged LOS included:<br>• Increasing age<br>• Female gender<br>• Karnofsky performance status<br>• Renal impairment<br>• History of dialysis<br>• Intubation, IABP, ventricular assist device or ECMO pre-transplant<br>• Previous cardiac surgery<br>Pulmonary hypertension
Goltzman et al<br><br>2019<br><br>Israel	275 general medical patients<br>1 March 2013 and 30 June 2013.<br>50.5% female<br>Mean age 68.3 $\pm$ 17.0 yrs<br>Single centre study	Prolonged hospitalisation defined as > 7 days	C-reactive protein was associated with the risk of prolonged hospitalisation with AUC for ROC of 0.73
Anderson et al<br><br>2015<br><br>USA	12511 general medical patients admitted 18363 times between Jan 2012 to Dec 2014	Prolonged LOS defined as > 21 days was observed in 2.3% of discharges<br><br>Prolonged hospitalisations accounted for 18.6% of total inpatient days	Prolonged hospitalisation was associated with<br>• Younger age<br>• MRSA septicaemia

	Single centre study<br><br>Mean age approx. 55 yr<br>Approx. 50% female		• Require surgery > 24 hr after admission<br>• Need for palliative care consultation
Barba et al<br><br>2015<br><br>Spain	All patients admitted under internal medicine to Spanish public healthcare sector 1st Jan 2005 to 31st Dec 2013<br><br>5275139 discharges<br><br>Multi-centre study<br>Mean age approx. 72 yr<br>52.5% male	Prolonged LOS was defined as > 30 days<br><br>3.2% had prolonged LOS but accounted for 17.4% of inpatient days<br><br>Did not adjust for multiple co-variates	Prolonged hospitalisation was associated with<br>• Younger age<br>• Male gender<br>• Elements of the Charlson co-morbidity index<br>• Palliative care consultation<br>• Surgery<br>• Discharge to a post-acute-care facility
Bateni et al<br><br>2018<br><br>USA	1928 patients with disseminated malignancy who underwent abdominal surgery for bowel obstruction<br>2007-2012<br><br>Multi-centre study<br><br>55% female<br>Mean age = 63 $\pm$13 years	Prolonged hospitalisation was defined as a LOS $\geq$ 75th centile ($\geq$ 20 days)	Prolonged LOS was ***not*** associated with:<br>• ASA status<br>• CCI score<br>Modified frailty index
Belkin et al<br><br>2019<br><br>USA	3021 patients who underwent thoracic endovascular aortic repair 2005-2015<br><br>2163 for aneurysm<br>858 for aortic dissection<br><br>Multi-centre study<br><br>Male gender comprised 60.5% dissection and 55.0% of aneurysm patients	Prolonged LOS was defined as > 75th centile (11 days)<br><br>Median post-operative LOS was<br>• 6 (3-11) days overall<br>• 7 (4-13) days for dissection<br>• 5 (3-10) for aneurysm	Variables associated with prolonged LOS<br><br>Dissection<br>• ASA score<br>• Days to operation<br>• Stroke / TIA<br>• Fail to wean > 48 hr<br>• Number of sepsis events<br>• Pneumonia<br>• Re-operation<br>Aneurysm<br>• Diabetes<br>• Dialysis<br>• Emergency surgery<br>• Days to operation<br>• Dependent status<br>• Cardiac history<br>• Transfusion required<br>• Stroke / TIA<br>• Fail to wean > 48 hr<br>• Number of DVT events<br>• Number of sepsis events<br>• Pneumonia<br>• Reintubation<br>• Postoperative AKI<br>• Re-operation
Carter et al<br><br>2015<br><br>USA	9593 laparoscopic bypass surgical operations in calendar year 2011<br><br>Multi-centre study<br><br>80% female<br>Mean age = 44 $\pm$ 12 years	Median LOS 2 days (range 0-544)<br><br>26% required $\geq$ 3 days hospitalisation	Longer hospitalisation was associated with:<br>• Diabetes<br>• Chronic obstructive pulmonary disease<br>• Bleeding disorder<br>• Increased serum Cr<br>• Low albumin<br>• Resident involvement in case<br>• Prolonged OR time<br><br>Longer hospitalisation was not associated with gender, age or BMI

Casillas-Berumen et al<br><br>2018<br><br>USA	1172 patients undergoing open abdominal aortic aneurysm repair 2012 – 2014<br><br>Multi-centre study<br><br>74% male<br>54% older than 70 years<br>Mean age = 70 ± 9 years	Above 75th centile (>10 days)	Prolonged LOS was associated with<br><br>• Visceral revascularisation<br>• Not from home admission<br>• Nature of the aneurysm<br>• Chronic obstructive pulmonary disease<br>• Age > 70 years
Dasenbrock et al<br><br>2015<br><br>USA	11,510 patients undergoing craniotomy for resection of brain tumour 2007-2013<br><br>Multi-centre study<br><br>55.8% aged > 56 yrs<br><br>53.2% female	Prolonged LOS > 75th centile (≥ 8 days)<br><br>Median (IQR) hospital LOS = 4 (3-8) days<br><br>27.7% had LOS ≥ 8 days	Prolonged LOS was associated with<br><br>• Age > 70 years<br>• ASA ≥ 3 designation<br>• Functional status<br>• IDDM<br>• Haematological co-morbidity<br>• Pre-operative hypoalbuminemia<br>• Post-operative complications<br>o Pulmonary embolism<br>o Pneumonia<br>o Urinary tract infection<br><br>Much of the variation in LOS was attributable to baseline co-variates suggesting it may not be modifiable
Dunn et al<br><br>2015<br><br>USA	2004 patients undergoing total shoulder arthroplasty 2005-2011<br>Multi-centre study<br><br>57 % women	Mean LOS after surgery = 2.2 (1.7) days<br><br>9% stayed ≥ 4 days	Los was treated as a continuous variable in the statistical analysis
Lakomkin et al<br><br>2019<br><br>USA	2170 patients undergoing surgical resection of spinal tumour between 2008 and 2014<br>54% were male<br>Mean aged was 57 ± 16 yrs<br>Multi-centre study	Prolonged LOS defined as above 75th centile	Prolonged LOS was associated with<br>• Charlson co-morbidity index<br>• Modified frailty index
Inneh et al<br><br>2015<br><br>USA	1486 patients undergoing total hip arthroplasty 2011-2014<br>Age range 21-93 years<br>Mean age 62<br><br>56% were females<br><br>Single centre study	Post-operative LOS ranged 1-27 days (mean 3.5 days)<br><br>32% had extended LOS > 3 days	Independent associations with prolonged LOS included:<br><br>• SES status<br>• Female gender<br>• Age ≥ 65 years<br>• Obesity<br>• ASA score > 2<br>• Length of surgery<br>• General anaesthesia<br>• Disorders of<br>o Circulatory system<br>o Respiratory system<br>o Genito-urinary system
Kaoutzanis et al<br><br>2015<br><br>USA	28269 cases of ventral/incisional hernia repair 2009-2010 in patients ≥ 18years<br><br>Mean age 55 years<br>51.6% female<br><br>Multi-centre study	25% had LOS ≥ 3 days<br><br>1% had LOS > 14 days	Independent associations with prolonged LOS included:<br><br>• Older age<br>• Functional status<br>• Alcohol abuse<br>• ASA status<br>• Prior operation in past 30 days<br>• History of chronic obstructive pulmonary disease<br>• History of congestive heart failure

			• Operation time<br>Emergency case
Lakomkin et al<br><br>2017<br><br>USA	6121 patients undergoing revision total hip arthroplasty Oct 2006 to Oct 2013<br><br>Mean age = 66 $\pm$ 13 years<br>55.3% female<br><br>Multi-centre study	Above 75th centile or at least 5 days	Prolonged LOS was associated with<br>• Female gender<br>• Operative time<br>• Functional dependence<br>• ASA score<br>• General anaesthesia<br>• Femoral revision<br>Charlson co-morbidity index
McGirt et al<br><br>2017<br><br>USA	6921 patients undergoing elective lumbar spine surgery<br><br>Patients > 18 years<br>Single centre study	Extended LOS was defined as $\geq$ 7 days<br><br>4.2% of patients required extended LOS	Prolonged LOS was associated with<br>• Non spinal fusion<br>• ASA classes<br>• Age<br>• Diabetes<br>• Independence with ambulation
Menendez et al<br><br>2015<br><br>USA	40869 patients undergoing elective arthroplasty<br><br>Age $\geq$ 18 years old<br><br>Jan – Dec 2011<br><br>Mean age approx. 70 years<br><br>Multi-centre study<br>Approx. 62% female	Prolonged LOS > 75th centile	Prolonged LOS was associated with<br>• Female<br>• Increasing age<br>• Congestive heart failure<br>• Renal failure<br>• Chronic obstructive pulmonary disease<br>• Pre-operative oedema<br>• Post-operative complications
Petis et al<br><br>2016<br><br>Canada	120 patients undergoing total hip arthroplasty<br><br>Mean age approx. 66 years<br><br>Single centre study<br>64.2% female	Prolonged LOS defined as > 48 hr<br><br>Mean LOS = 54.3 $\pm$ 26.4 hr	Prolonged LOS was associated with:<br>• Time to up and go<br>Prolonged LOS was not associated with<br>• Age<br>• Body mass index<br>• Mean procedure time<br>Mean time in post-anaesthetic care
Velagapudi et al<br><br>2018<br><br>USA	678545 hospitalisations for primary percutaneous coronary intervention to ST-elevation myocardial infarction between 2005 to 2014<br><br>Multi-centre study<br><br>Mean age = 59.6 yrs<br><br>25.4% females	Median (IQR) LOS = 3.0 (2.0-3.0) days<br><br>LOS > 3.0 days occurred in 24.1%	Prolonged LOS was associated with:<br>• Age $\geq$ 65 years<br>• Female gender<br>• Hospital size and location<br>• Heart failure<br>• Peripheral arterial disease<br>• Chronic lung disease<br>• Chronic kidney disease<br>• Atrial fibrillation<br>• Coagulopathy<br>• Depression<br>• Anterior STEMI<br>• stroke<br>• coagulopathy / bleeding<br>vascular complication
Quintana et al<br><br>2015<br><br>Spain	3276 episodes of exacerbation of chronic obstructive pulmonary disease between June 2008 and Sep 2010<br><br>Multi-centre study	Prolonged LOS defined as > 9 days	Prolonged LOS was associated with:<br>• FEV1 < 50<br>• Admission in past 12 months<br>• Prior home oxygen or non-invasive ventilation<br>• Number of treatments<br>• Baseline physical activity<br>• ICU admission<br>• $PaCO_2$ > 65 vs $\leq$ 45 mmHg<br><br>Prolonged LOS was not associated with<br>• age $\geq$ 70 years<br>• gender<br>• CCI<br>• Intravenous antibiotics<br>Diuretics

Wang et al<br><br>2017<br><br>USA	334 patients undergoing lower limb vascular bypass procedures between 2010 and 2015<br><br>Single centre study<br><br>Mean age 66.4 ± 12.4 yr<br>64.7% males	Mean LOS = 15.7 ± 12.2 days<br><br>Prolonged LOS was defined as > 7 days<br>247 (74.0%) had prolonged LOS<br><br>The LOS group stayed on average approximately 2 weeks longer than the non-prolonged LOS group (19.7 ± 12.3 vs. 4.9 ± 1.6 days, respectively, P < 0.001)	Prolonged LOS was associated with:<br>• Urgent surgery<br>• Critical limb ischemia<br>• Return to the operating room<br>• Use of a vein graft<br>• The need for anticoagulation at discharge
Winemaker et al<br><br>2015<br><br>Canada	1459 patients undergoing elective total knee (61.7%) and total hip (38.3%) replacement between Apr 2011 and Mar 2012<br>Single centre study<br><br>57.5% female<br>Median age = 67 (52-82) yr	LOS was characterised as ≤ 3, 4, or ≥ 5 days<br><br>516 (35.4%) stayed ≥ 5 days	Longer LOS was associated with:<br>• Female gender<br>• Hip > knee replacement<br>• Age ≥ 75 years<br>• ASA 3 or 4<br>• Number of cardio-vascular and renal co-morbidities<br>Current smoker
Zhan et al<br><br>2019<br><br>China	303 patients aged ≥ 60yr undergoing first time elective open posterior lumbar fusion between Dec 2012 and Dec 2017<br>Single centre study<br><br>Mean age = 67.0 ± 5.5 yr<br><br>54.8% were men	Mean LOS = 18.5 ± 11.8 days<br>Range = 7-103 days<br><br>83 (27.4%) had an extended LOS (≥ 75th centile)	Extended LOS was associated with:<br><br>• Age<br>• Pre-operative waiting time<br>• Pulmonary complications<br>• Diabetes<br>• Intra-operative blood loss<br>• Multi-level procedure<br>• Major complications<br>• Infectious complications<br>• Multiple surgeries<br>Surgical bleeding
